# On how the binding cavity of AsqJ dioxygenase controls the desaturation reaction regioselectivity: a QM/MM study

**DOI:** 10.1007/s00775-018-1575-3

**Published:** 2018-06-06

**Authors:** Zuzanna Wojdyla, Tomasz Borowski

**Affiliations:** Jerzy Haber Institute of Catalysis and Surface Chemistry, Polish Academy of Sciences, Niezapominajek 8, 30239 Kraków, Poland

**Keywords:** ODD, AsqJ dioxygenase, 4′-methoxyviridicatin, Hydrogen atom abstraction, C–H bond activation, Desaturation/hydroxylation selectivity, QM/MM, DFT

## Abstract

**Abstract:**

The Fe(II)/2-oxoglutarate-dependent dioxygenase AsqJ from *Aspergillus nidulans* catalyses two pivotal steps in the synthesis of quinolone antibiotic 4′-methoxyviridicatin, i.e., desaturation and epoxidation of a benzodiazepinedione. The previous experimental results signal that, during the desaturation reaction, hydrogen atom transfer (HAT) from the benzylic carbon atom (C10) is a more likely step to initiate the reaction than the alternative HAT from the ring moiety (C3 atom). To unravel the origins of this regioselectivity and to explain why the observed reaction is desaturation and not the “default” hydroxylation, we performed a QM/MM study on the reaction catalysed by AsqJ. Herein, we report results that complement the experimental findings and suggest that HAT at the C10 position is the preferred reaction due to favourable interactions between the substrate and the binding cavity that compensate for the relatively high intrinsic barrier associated with the process. For the resultant radical intermediate, the desaturation/hydroxylation selectivity is governed by electronic properties of the reactants, i.e., the energy gap between the orbital that hosts the unpaired electron and the sigma orbital for the C–H bond as well as the gap between the orbitals mixing in transition state structures for each elementary step.

**Graphical abstract:**

Regiospecificity of the AsqJ dehydrogenation reaction is dictated by substrate–protein interactions. 82 × 44 mm (300 × 300 dpi)
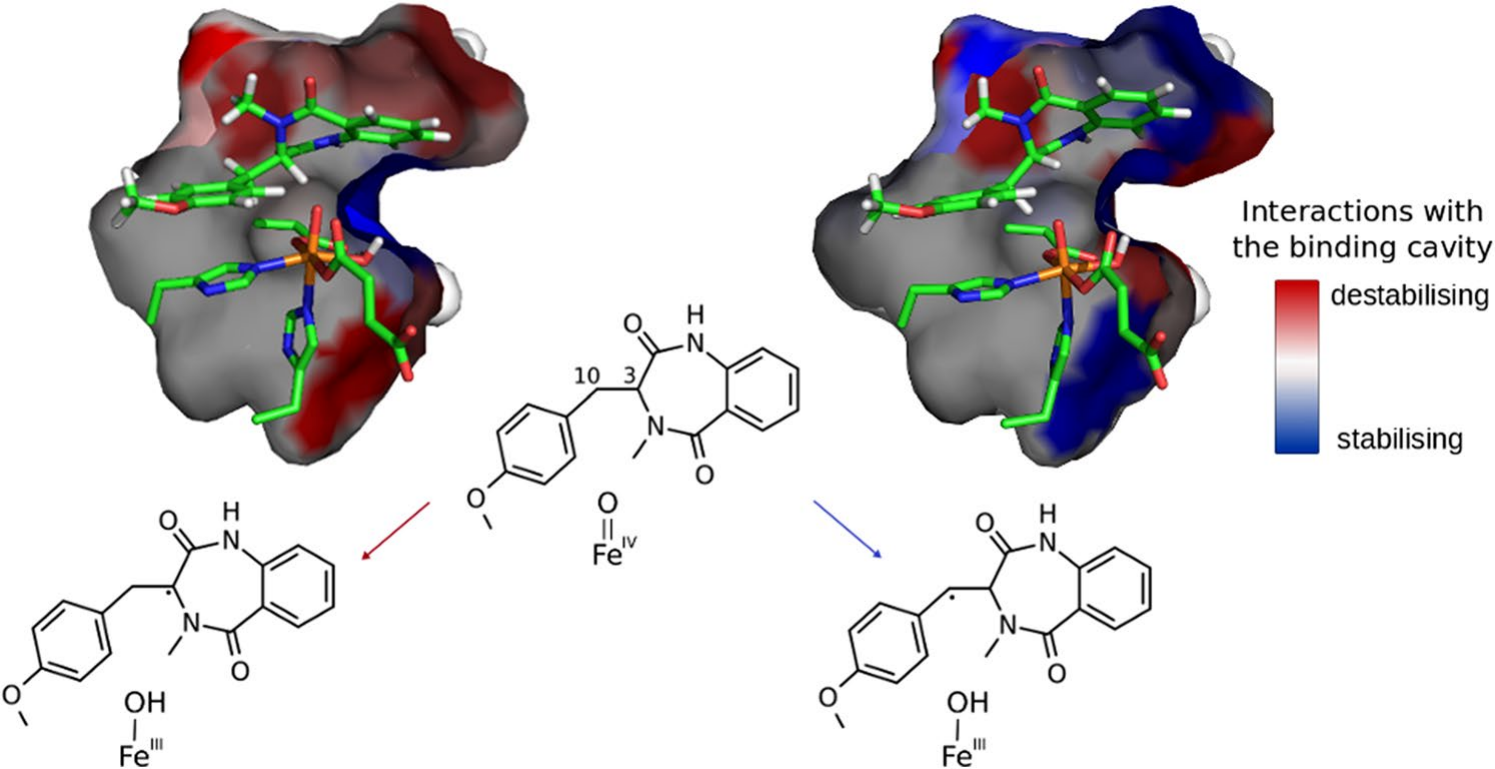

**Electronic supplementary material:**

The online version of this article (10.1007/s00775-018-1575-3) contains supplementary material, which is available to authorized users.

## Introduction

The Fe(II)/2-oxoglutarate-dependent dioxygenases (ODD) are mononuclear enzymes that catalyse a wide variety of reactions, i.e., hydroxylations, desaturations, halogenations, or cyclisations [1, 2]. These oxidative transformations are initiated by reactive Fe(IV)-oxo (oxoferryl) species formed when 2-oxoglutarate (2OG) is oxidatively cleaved into succinate and CO_2_ with use of molecular oxygen [3]. A typical iron-binding site of the ODD is composed of three amino acids from the HXDX_*n*_H triad motif characteristic for the family [4], 2OG bound to Fe(II) in the bidentate mode, and a water molecule [5].

**Fig. 1:**
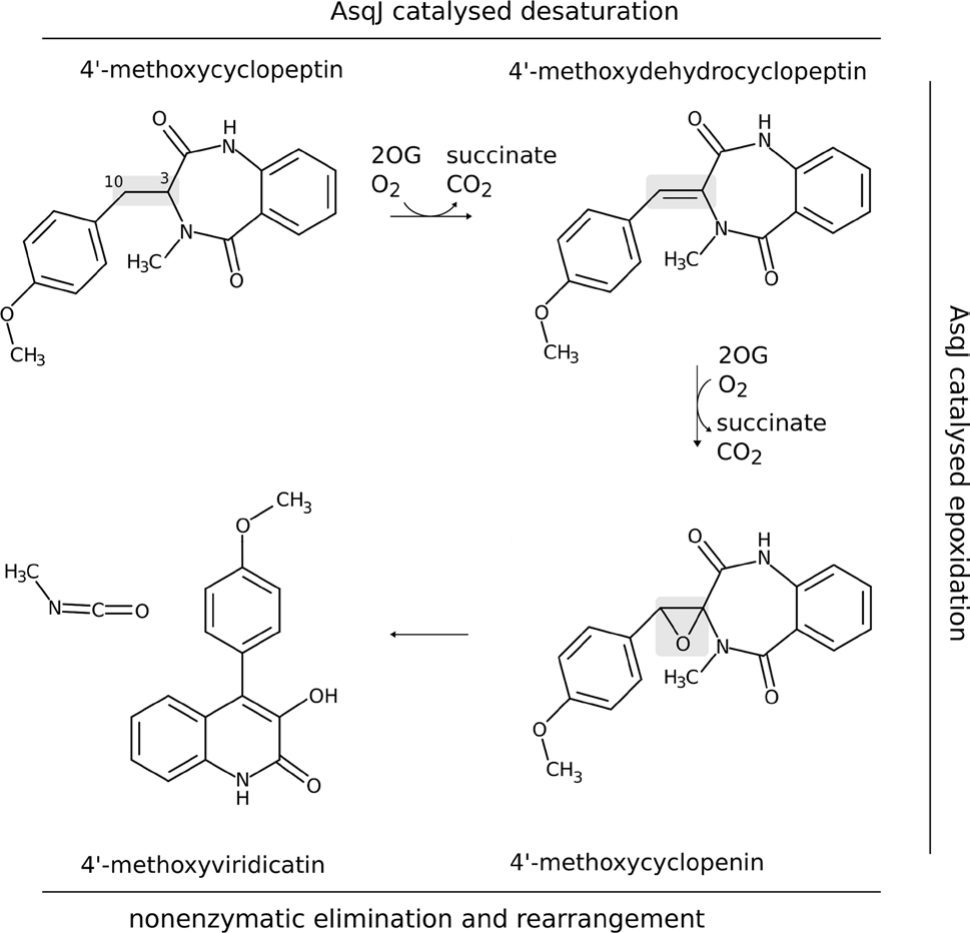
Desaturation and epoxidation reactions catalysed by AsqJ and the final nonenzymatic rearrangement. The oxidised fragment is indicated in grey. Marvin was used for drawing chemical structures [18]

Dioxygenase AsqJ from *Aspergillus nidulans* catalyses two key steps in biosynthesis of a quinolone antibiotic. First, the substrate, 4′-methoxycyclopeptin, is desaturated to form 4′-methoxydehydrocyclopeptin (see Fig. 1), which is, later, in a second reaction catalysed by AsqJ, epoxidized at the same position [6]. The resultant epoxide departs from the binding cavity to the solution where it undergoes an equally fascinating acid/base catalysed rearrangement leading to the tricyclic quinolone antibiotic molecule, 4′-methoxyviridicatin [7]. The recent experimental results suggest that desaturation is initiated by hydrogen atom transfer (HAT) at the benzylic (C10) position by the high-spin oxoferryl species (consult Fig. 1) and, once the radical/Fe(III)-OH intermediate is formed, it might proceed through the hydroxylated or carbocation intermediate [8] or via a second HAT from the C3 atom from the ring moiety. Results of a previous QM/MM study on the reaction mechanism of AsqJ suggested that the energetically favourable desaturation pathway involves HAT at the C3 position (with energy barrier of 19.3 kcal mol^−1^), whereas the alternative HAT from C10 is hindered by a high barrier of 30 kcal mol^−1^ [9]. This result is at odds with the experimental findings, which indicate the opposite regioselectivity of the initial HAT process [8]. The differences could stem from the choice of Fe(IV) coordination geometry that was assumed in the computational study. More specifically, the previous joint experimental and computational study on the activation of AsqJ revealed that the metal ion in the oxoferryl complex is hexacoordinated with one water molecule occupying the equatorial position and another water molecule in a second coordination shell [10]. In contrast, in the QM/MM study on the desaturation mechanism, a pentacoordinated complex in trigonal bipyramidal geometry was used. In such a model, the oxo ligand was located far from the substrate (trans to His-134) and, consequently, the computed reaction energy profiles might not reflect the actual interactions within the AsqJ active site. Therefore, it seems pertinent to revisit the mechanism of the desaturation reaction with QM/MM methods for an experimentally consistent geometry of the activated metal cofactor and to address the still open questions concerning product specificity and regioselectivity of the reaction.

The experimentally observed reaction outcome is the dehydrogenated product [6], which raises a question about the roots of selectivity in a system that can facilitate both desaturation and hydroxylation. The selectivity of the ODD catalysed reactions is a widely investigated yet still puzzling issue. Results of a hybrid DFT study on the cluster model of TauD and a synthetic shape-selective catalyst (TpOBzFe) indicated that the switch between the desaturation and hydroxylation channels depends on intrinsic factors such as C–H bond strength and delocalisation of the unpaired electron on the radical intermediate; moreover, a gap between the electron donating orbitals, i.e., σ_C–H_ (operative in desaturation) and φ_C_ (operative in hydroxylation) alongside energetic proximity of these orbitals to the acceptor one ($\pi_{xz/yz}^{*}$), plays a major role in reaction selectivity [11]. Apart from this energetic factor, the extent of overlap between the donor and acceptor orbitals can promote either radical (OH) rebound or HAT. It was shown for synthetic manganese catalysts that relative orientation of the radical and the Mn(IV)–OH moiety governs the desaturation/hydroxylation partitioning [12]. Flexibility of the substrate radical and steric accessibility of the active site was also shown to be a factor that favours the desaturation pathway in non-heme diiron hydroxylase AlkB [13]. Similarly, the 2-oxoglutarate-dependent halogenase, SyrB2, controls the hydroxylation/halogenation selectivity by positioning the fragment of the molecule that host the unpaired electron closer to the halogen ligand that to the hydroxyl moiety [14, 15]. In the later study, it was shown, using frontier molecular orbital arguments, that even though hydroxylation is thermodynamically favoured, chlorination is more accessible due to lower energy of the orbital that accepts the electron in the halogenation reaction, i.e., ${\text{d}}\pi_{{{\text{Fe}}{-}{\text{Cl}}}}^{*} .$ [16].

In addition, desaturation versus hydroxylation bifurcation was investigated for heme-containing cytochrome P450 with the use of valence bond theory. This study revealed that the preference for a given reaction stems from intrinsic properties of the reactants, i.e., energies of the bonds formed in the competing processes (O–H and O–C) energy of the C–H bond and the π-conjugation energy. Interestingly, comparison of QM and QM/MM results indicated that the electronically driven preference can be altered by steric constraints imposed by the enzyme-binding pocket [17].

The aim of this study was to elucidate the origins of HAT regioselectivity as well as unravel the factors that determine the preference for desaturation over hydroxylation displayed by AsqJ. The beneath reported computational results show that the reaction is initiated by HAT from the C10 atom and the preference for this pathway stems from favourable interactions between the substrate molecule and the binding cavity of AsqJ in the relevant transition structure. In the energetically favoured path, the selectivity for desaturation over hydroxylation is rooted in a smaller energy gap between electron donating and electron accepting orbitals for the former process. This inherent property is not compensated by the protein environment, but the analysis of protein–ligand interactions indicates that the AsqJ reaction selectivity could be changed by appropriate amino acid substitutions.

## Computational methods

### Model preparation and MD simulation

The model of the dimeric protein was constructed based on crystal structure for Ni^2+^-substituted AsqJ with 2OG and 4′-methoxycyclopeptin bound in the active site (PDB ID: 5DAQ) [7]. To obtain the model of the active protein, Ni^2+^ was replaced with Fe^2+^. Hydrogen atoms were added with the LEaP program from the AmberTools 14 package [19] and the system was solvated by placing it in a box of TIP3P water [20] with the minimal distance between the atoms of the protein and the wall of the box equal to 10 Å. Water molecules present in the crystal structure were retained. Charge of the protein was neutralised with 4 Na^+^ ions. The minimisation and MD simulation were performed with use of Amber ff03 force field [21]. Missing parameters for residues ligating Fe^2+^ were calculated based on the model of the first coordination shell of Fe^2+^, i.e., His-134, Asp-136, and His-211, 2OG, and water molecule. The backbone of the ligating amino acids was truncated at the Cα of the neighbouring residues. The model was optimised with the spin-unrestricted B3LYP-D3 [22, 23] method and LanL2DZ basis set [24]; the electrostatic potential (ESP) was calculated with Gaussian 09 [25] at the B3LYP/cc-pVTZ/IEFPCM (*ε* = 4, *r* = 1.4 Å) level [26]. Atomic charges of the residues were calculated with use of restrained electrostatic potential (RESP) formalism as implemented in antechamber [27].

Prior to molecular dynamics (MD) simulation, the system was minimised in three steps. First, the solvent molecules were optimised, whereas positions of all atoms of the solute were restrained with a 500 kcal mol^−1^ harmonic potential. In the next step, the restraining potential was reduced to 10 kcal mol^−1^. The final minimisation was performed without restrains for the whole solute except the first coordination shell of Fe^2+^, which was fixed with a 500 kcal mol^−1^ potential to retain the geometry consistent with the one from the crystal structure. These restraints were present throughout all minimisation and MD steps. Such an approach is commonly used to treat non-heme iron containing enzymes [28–30]. MD simulation started with heating of the system from 0 to 300 K in 100 ps followed by 1 ns-long density equilibration (NTP). The MD production run was performed for 10 ns in constant temperature equal to 300 K and a constant pressure equal to 1 atm under periodic boundary conditions. Time step was equal to 2 fs and all bonds involving hydrogen atoms were constrained with use of the SHAKE algorithm [31]. The electrostatic energy of a cell was calculated by the particle-mesh Ewald method [32]. The system reached equilibrium after 2.5 ns, as the root-mean-square displacement (RMSD) of the backbone of the protein revealed and the stable part of the trajectory (2–10 ns, consult Fig. S1) was further clustered with use of agglomerative method as implemented in CPPTRAJ [33]. The representative of the dominant cluster was optimised with the ONIOM method with Gaussian 09 (vide infra).

The active site of the optimised structure was modified in the following way: 2OG was replaced with succinate positioned in the same plane, oxo ligand was located in axial position trans to His-211 and water molecule was added as an equatorial ligand trans to His-134, thus forming octahedral geometry as reported previously for AsqJ [10]. The geometry of the system was optimised again with Gaussian 09 and prepared for MD simulation in the same way as the AsqJ:2OG complex. The bonded parameters involving Fe were derived based on calculations for a bigger model that incorporated three additional residues (Gln-131, Thr-172, and Arg-223) necessary to retain the orientation of succinate. The production run of the MD was elongated to 21 ns and the representative snapshot for the QM/MM calculations was chosen based on clustering of the stable part of the trajectory (7–21 ns, the RMSD plot is shown in Fig. S2).

### ONIOM calculations

The QM/MM calculations were performed with the ONIOM method in the Gaussian 09 program [25] on a model that consisted of the protein and all water molecules within 20 Å of the Fe ion. Positions of residues with atoms located further than 15 Å from the ion were fixed. The MM region was described with the Amber force field as implemented in Gaussian [34]. The QM part consisted of Fe(IV)-oxo species, side chains of residues ligating Fe(IV) (His-134, Asp-136, and His-211), equatorial water molecule, and succinate modelled by acetate. Geometries were first optimised with the spin-unrestricted B3LYP method [22] and the def2-SVP basis set [35–37] employing the mechanical embedding scheme. To take into account the impact of the partial charges of the MM region on the QM part, the re-optimisation procedure was employed. First, the ESP of the QM part was calculated at the same level of theory as the optimisation was performed; MM part was modelled as point charges; however, the link atoms and their nearest (bonded) neighbours were omitted. The new atomic charges of the QM part were obtained with the RESP procedure. The structures were re-optimised with the new atomic charges at the B3LYP-D3/def2-SVP level [23] with Gaussian 16 [38], which was followed by frequency calculation. Finally, single-point energy calculations for stationary points were performed with the same functional and the def2-TZVP basis set [36] with mechanical and electronic embedding. The reported energy values, unless stated otherwise, are ONIOM(B3LYP-D3/def2-TZVP, Amber) energies computed with electronic embedding plus Gibbs free energy corrections obtained at the ONIOM(B3LYP-D3/def2-SVP, Amber) and mechanical embedding level. The reported orbital gap energies as well as distortion energies were calculated using the B3LYP-D3/def2-TZVP method for the QM part of the system (or its fragments).

### Alanine screening

In the structures of the mutant proteins, side chain of a chosen amino acid was replaced with the methyl group. Single-point MM energy calculations for the structures ($E_{\text{MM}}^{\text{mut}}$) were performed with Amber force field in Gaussian 16. As the change is restricted to the MM part of the system, values of MM ($E_{\text{MM}}^{\text{M}}$) and QM energy ($E_{\text{QM}}^{\text{M}}$) of the model system were the same as in the respective optimised wild-type structures. The ONIOM energies of the ES and TS structures for the mutant were calculated following the subtracting coupling scheme:

$$E_{{{\text{QM}}/{\text{MM}}}}^{\text{mut}} = E_{\text{MM}}^{\text{mut}} - E_{\text{MM}}^{\text{M}} + E_{\text{QM}}^{\text{M}} ,$$

where $E_{\text{MM}}^{\text{mut}}$ stands for single-point MM energy of a mutant structure. $E_{\text{QM}}^{\text{M}}$ is a value calculated at the B3LYP-D3/def2-TZVP level with mechanical embedding for the QM region of wild-type structure. In the further analysis, the barrier heights, computed as E(TS)-E(ES), were compared with those obtained for the wild-type form of the enzyme to assess the stabilisation/destabilisation effect of the chosen residue.

### Cluster calculations

The cluster calculations were performed on a model involving the iron ion and its first coordination shell (His-134, Asp-136, His-211, water molecule, acetate, and the oxo ligand) and the substrate molecule. The positions of the Cβ and the methyl C of acetate were fixed during optimisations. Optimisations and frequency calculations were performed with the spin-unrestricted B3LYP and def2-SVP basis set [22, 36]. Solvent corrections for the optimised geometries were calculated at the same level of theory and the IEFPCM model [39] with *ε* = 4.0 to model the protein environment. Energy was also calculated in a single-point manner with def2-TZVP basis set. The reported energy is B3LYP-D3/def2-TZVP energy with ZPE and solvent corrections.

### QM calculations for the nonenzymatic reaction

The X-ray structure of 3′-hydroxycyclopenin was obtained from the CSD [40] (CSD reference code POHBEV [41]), the C3′-bound hydroxyl group was replaced by hydrogen atom and methoxy group was introduced at the C4′ position to construct 4′-methoxycyclopenin. The Brønsted base and acid compounds that take part in the reaction were modelled as acetate and acetic acid or ascorbate and ascorbic acid. The geometry optimisations and analytical frequency calculations were performed at the B3LYP-D3/6-31G level [42] with solvent (water) modelled using the IEFPCM model with dielectric constant of the homogeneous dielectric medium equal to 78. For stationary points, single-point energy calculations were performed with the 6-311G(d,p) basis set [43] and PCM model. The reported energy values are B3LYP-D3/6-311G(d,p) energies with Gibbs free energy corrections.

## Results and discussion

### Enzymatic reaction

#### Enzyme–substrate complex: MD approach

**Fig. 2:**
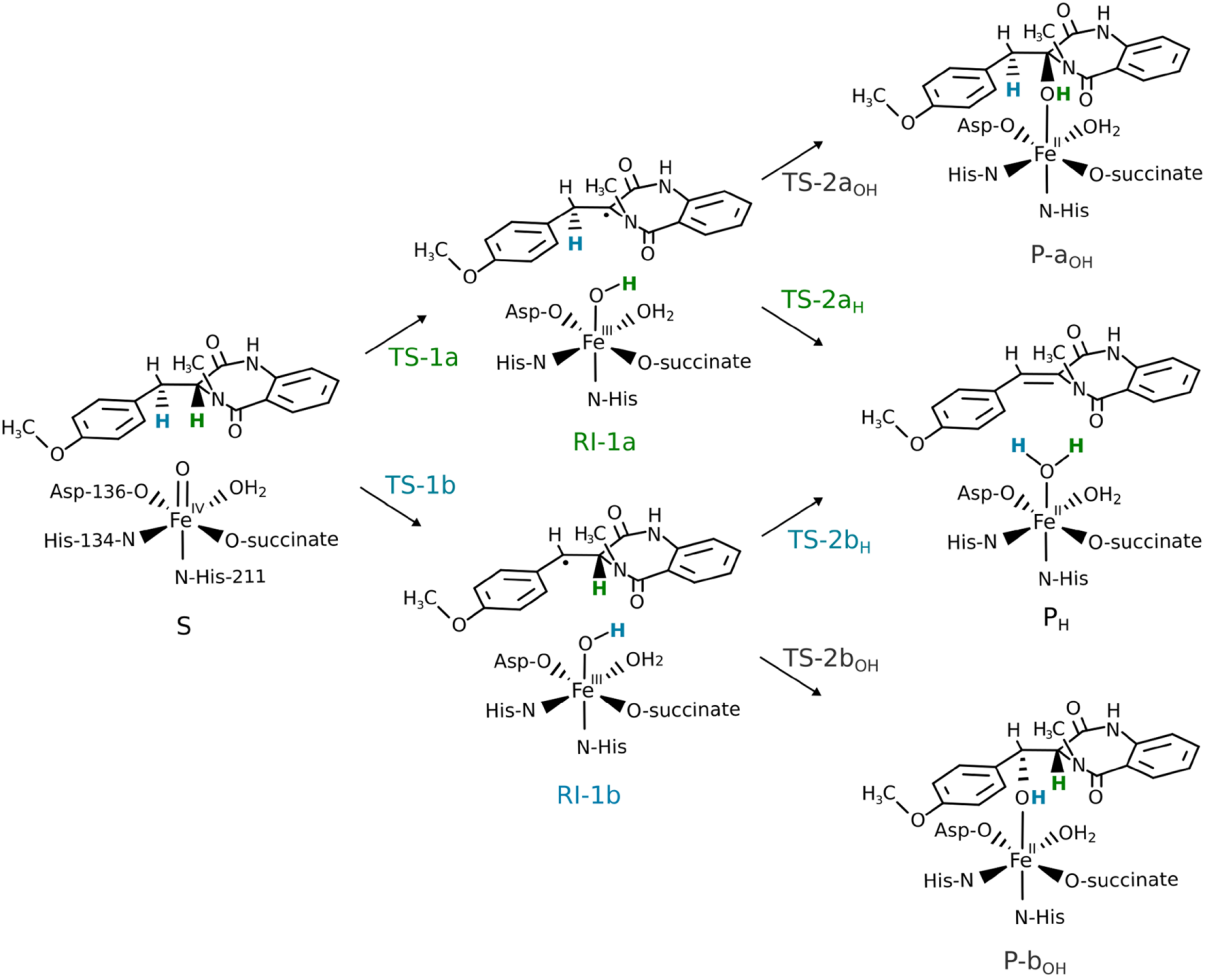
Possible pathways for AsqJ-catalysed oxidation reactions of 4′-methoxycyclopeptin. C3-bound hydrogen is shown in green; C10-bound hydrogen in blue. Marvin was used for drawing chemical structures [18]

In the first step of the desaturation reaction, the hydrogen atom could be abstracted from either the benzylic position (C10) or from the C3 atom of the bicyclic ring (see Fig. 2). In the crystal structure [7] (with hydrogens added with LEaP; see Computational Methods), the distance between the C3- and C10-bound hydrogen atoms and the oxygen atom of the carboxylate group of 2OG is 2.24 and 3.14 Å, respectively. The relevant Fe–O–H angles total to 113° and 77°. During the MD simulation, the substrate moves slightly away from the metal cofactor (consult Table S1 and Fig. S3A, B), and in the subsequently optimised ONIOM structure, the distance between the C3-bound or the C10-bound H and the O atom totals to 2.43 and 3.38 Å, respectively. In the optimised structure of the activated form, i.e., with 2OG substituted by succinate and Fe(II) oxidised to Fe(IV)=O, the distance between the reactants shortened by ca. 0.2 Å (for the C3-bound H) and by 0.6 Å (for the C10-bound H) and the respective Fe–O–H angles increased to 148° and 92° (Fig. S3C, D). During the subsequent MD simulation, all of the monitored values increased slightly. Snapshots of the final 11 ns-long part of the trajectory were clustered. In this procedure, the distance between frames was calculated as RMSD for the fragment of the substrate molecule that takes part in the reaction. The frames were divided into four clusters (the Davies–Bouldin Index: 0.69, the pseudo-*F* statistic: 60.82; clustering metrics obtained for three and five clusters were of lower quality). The dominant cluster covered 95.3% of the analysed trajectory; the average distance between points in the cluster was 0.068 (the average distance to centroid was 0.048) with standard deviation of 0.3, whereas the distance between this cluster and the other three was 0.125.

#### Hydrogen atom transfer

The optimised enzyme–substrate complex (**S**) can be described as a high-spin Fe(IV) (spin population of 3.18) coordinated by an oxo ligand (spin population of 0.59) and side chains of His-134, Asp-136, His-211, succinate, and a water molecule in octahedral geometry. Such a complex is the most likely species to initiate the oxidation of the primary substrate, as the previous Mössbauer and QM/MM study on AsqJ has revealed [10].⁠

In the optimised structure, the C3- and C10-bound hydrogens are positioned, respectively, 2.13 and 2.73 Å away from the oxo ligand of the oxoferryl species (Fig. S3F). Such close contacts are expected to facilitate hydrogen atom transfer (HAT) from both C10 (**TS-1b**) and C3 (**TS-1a**); however, values of the Fe–O–H angle (146° for C3-bound H, 90° for C10-bound H) suggest that HAT from C3 may be preferred, at least for the sigma channel, which usually requires angles larger than 120° [44–46].

**Fig. 3:**
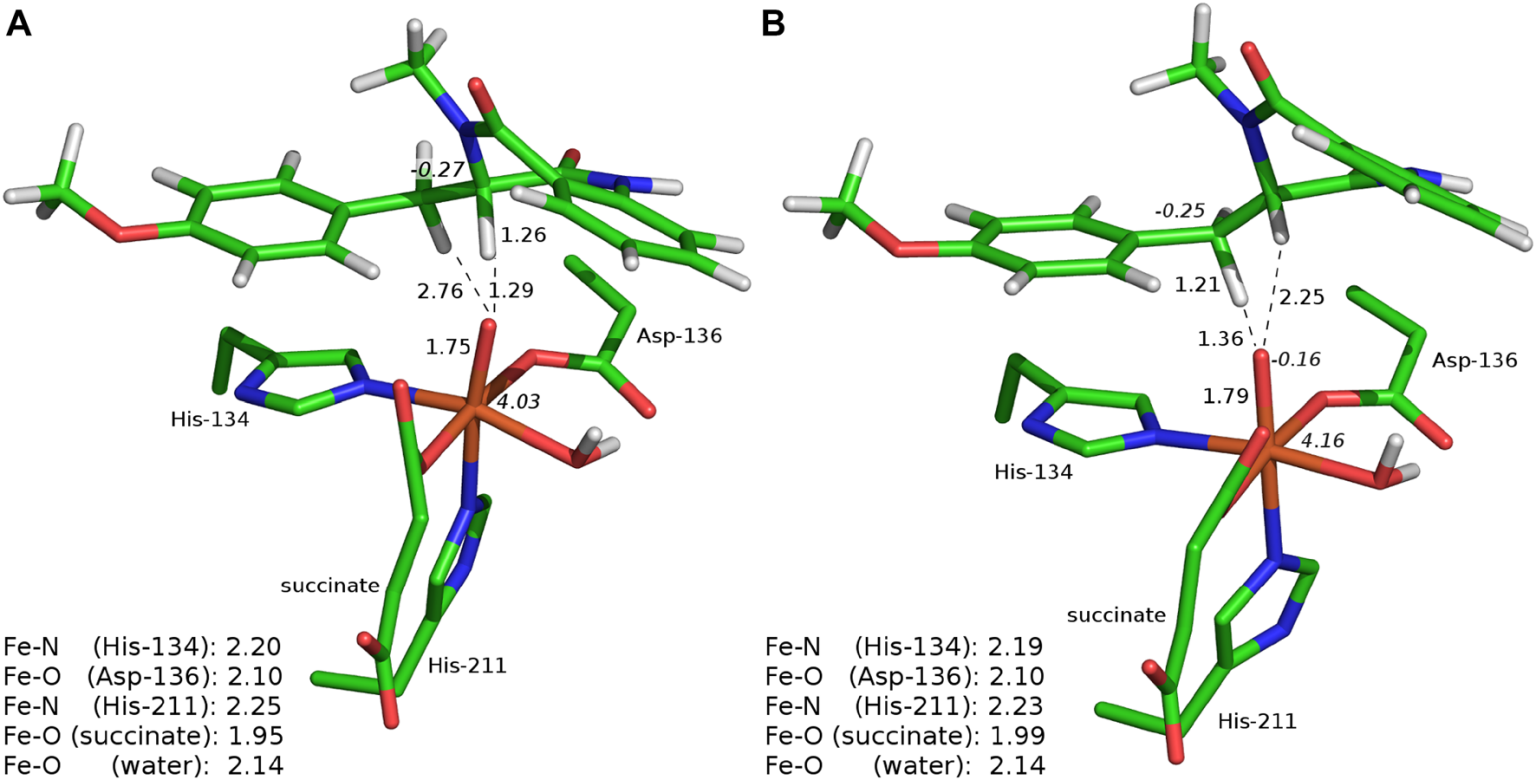
Optimised structures for **TS-1a** (**a**) and **TS-1b** (**b**). Distances are given in Å and spin populations larger than 0.1 are given in italics. Figure rendered with PyMOL [47]

**Fig. 4:**
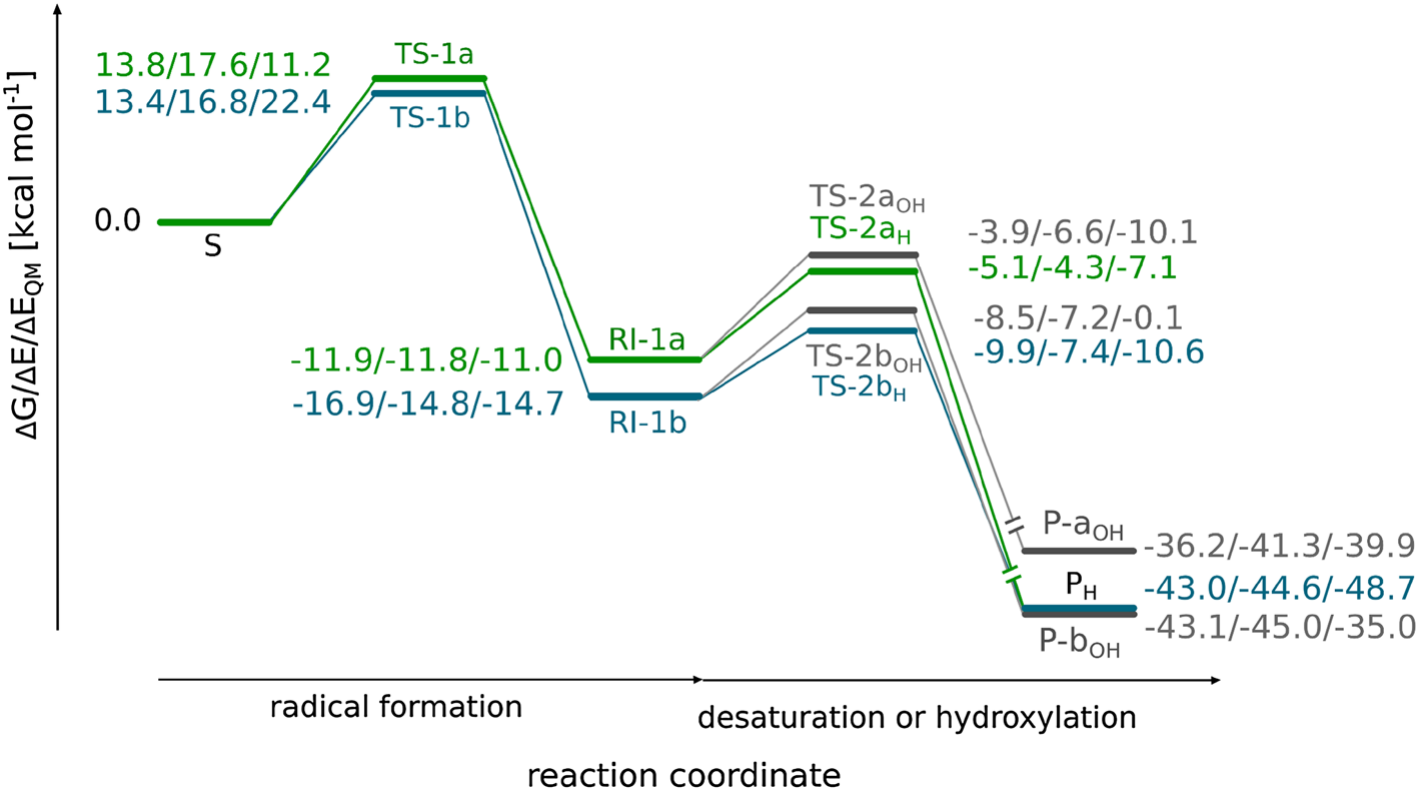
Reaction energy profile for AsqJ-catalysed desaturation and plausible hydroxylation. The energy values, i.e., Gibbs free energy (Δ*G*), relative potential energy calculated at the ONIOM(B3LYP-D3/def2-TZVP, Amber) level with electronic embedding and the relative potential energy of the QM part obtained with the B3LYP-D3/def2-TZVP method are given in kcal mol^−1^

The transition state structure associated with HAT at the C3 position (**TS-1a**) starts the σ-pathway as manifested by spin populations: 4.03 on Fe and − 0.27 on C3. For **TS-1a,** the Fe–O distance is slightly elongated as compared to **S**, 1.75 vs 1.65 Å (see Fig. 3a). The distance between the C3-bound hydrogen and the oxygen atom of oxoferryl species (1.29 Å), as well as the Fe–O–H angle (140°) are within a typical range for TS of HAT proceeding in the sigma channel (consult natural orbitals for spin density presented in Fig. S4A, B). The computed Gibbs free energy barrier for this process equals to 13.9 kcal mol^−1^ (Fig. 4). Formation of the radical intermediate (**RI-1a**) is exoergic by 11.9 kcal mol^−1^ and the spin density is located mostly on C3 (− 0.70) and high-spin Fe(III) (4.27). The Fe-bound hydroxide forms a hydrogen bond with the carboxyl group of succinate.

The Gibbs free energy barrier associated with the alternative route, passing through **TS-1b,** is by 0.4 kcal mol^−1^ lower than the barrier connected with **TS-1a** (see Fig. 4). This is in line with the results of a recent stopped-flow/UV–Vis study of AsqJ-catalysed desaturation, which suggest that the reaction is most likely initiated by abstraction of the C10-bound hydrogen by the Fe=O core [8]. Our computational results suggest that the first HAT is the rate-limiting step with a free energy barrier of 13.4 kcal mol^−1^ (**TS-1b**). In this transition structure, the distance between the hydrogen atom and the oxygen of the oxoferryl species totals to 1.36 Å and the Fe–O–H angle is equal to 114°. Typically, for such a sharp angle, the electron is expected to be transferred from the σ(C–H) to π*(Fe=O) orbital (π-pathway), as their overlap is significantly better than for the σ(C–H) and σ*(Fe=O) orbitals (σ-pathway) [48]. The spin population on C10 is − 0.25 and the Fe(III) ion is in the high-spin state (spin density totals to 4.16) (Fig. 3). Examination of natural orbitals for spin density (see Fig. S4C, D) indicates a transfer of the α electron between the C–H bond and the π*(Fe=O) orbital. It is consistent with the π-pathway involving high-spin Fe(III) (*S* = 5/2) and a β radical intermediate, which occurs alternatively to the π channel leading to intermediate-spin Fe(III) and is possible due to mixing of the electronic states enabled by breaking the symmetry of the system [49]. The TS associated with the π-pathway with Fe(III) (*S* = 3/2) lies ca. 5 kcal mol^−1^ higher in energy. The resultant intermediate (**RI-1b**) is a radical with the unpaired electron (β spin) delocalised over the C10 atom (− 0.68) and the anisyl ring (total spin population on the ring is − 0.27). The spin delocalisation most likely contributes to the stability of the intermediate, which lies 16.9 kcal mol^−1^ lower in energy than the initial reactant complex **S**.

#### Regioselectivity of HAT

The regioselectivity of HAT can be analysed with the use of Marcus theory [50] to obtain intrinsic barriers, i.e., ones unaffected by thermodynamic contributions. Such an approach was successfully employed by Srnec et al. to analyse the reaction selectivity of SyrB2 [16]. The relation between the observed $\Delta G^{{\ddag }}$ barrier and the intrinsic $\Delta G_{\text{int}}^{{\ddag }}$ is given by the equation:

$$\Delta G^{{}} = \Delta G_{\text{int}}^{{\ddag }} + \frac{{\Delta G_{0} }}{2} + \frac{{\Delta G_{0}^{2} }}{{16\Delta G_{\text{int}}^{{\ddag }} }} ,$$

where Δ*G*_0_ is the reaction energy, $\Delta G^{{\ddag }}$ and Δ*G*_0_ are known from the reaction-free energy profile. Thus, calculated intrinsic barrier associated with HAT from C3 (**TS-1a**; 19.4 kcal mol^−1^) is by 1.6 kcal mol^−1^ lower than the one for HAT from C10 (**TS-1b**; 21.0 kcal mol^−1^). These values indicate that the observed reaction preference is driven by higher thermodynamic driving force for the formation of **RI-1b** as compared to **RI-1a**.

Moreover, the preference for **TS-1b** over **TS-1a** can be observed only when interactions with the binding pocket are included in the computational model. The picture is different when the QM energies of these transition structures are analysed; in this case, the preferred process is HAT at the C3 position with electronic energy barrier equal to 11.2 kcal mol^−1^ (see Fig. 4). The electronic energy barrier associated with **TS-1b** is relatively high, it totals to 22.4 kcal mol^−1^ and its height is most likely caused by the excitation required to enter the π channel with *S*(Fe(III)) = 5/2.

**Fig. 5:**
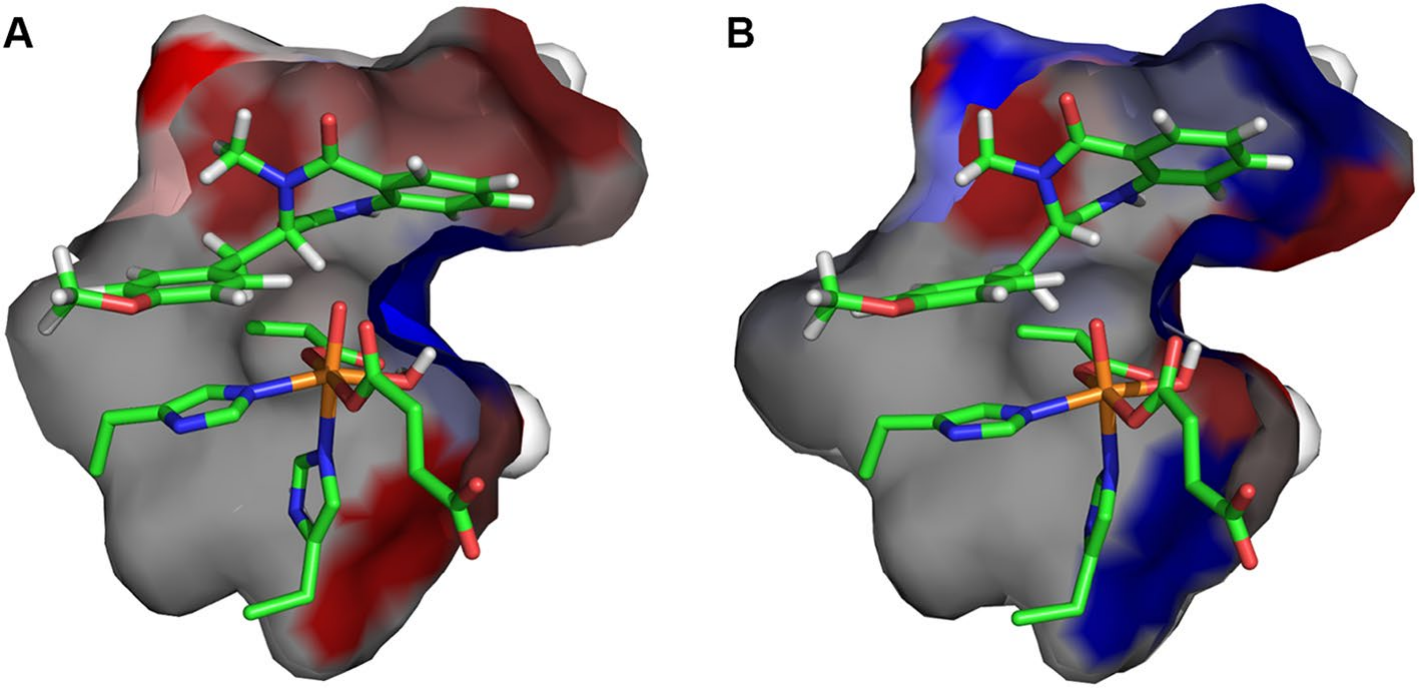
Binding cavity of AsqJ for **TS-1a** (**a**) and **TS-1b** (**b**). The amino acids lining the cavity are coloured from blue (stabilising the transition state) to red (destabilising the transition state)

To investigate the effect of protein environment for the HAT barriers, the amino acid residues with atoms within 5 Å of the substrate and 2OG molecule were consecutively replaced by alanine (alanine screening; for details of the procedure, see Computational methods). For such mutants, the ONIOM energy of **S**, **TS-1a**, and **TS-1b** was recalculated in a single-point manner. As shown in Fig. 5b, the positioning of the substrate in optimised structure of **TS-1b** allows for favourable (mostly) interactions with hydrophobic residues lining the cavity. Comparison of optimised structures of **TS-1a** and **TS-1b** reveals that, in **TS-1a,** the substrate moves slightly away from the roof (upper part; as shown in Fig. 5a) of the binding pocket, which weakens interactions with hydrophobic residues (for details, see Figs. S5, S6, and Table S2), but this movement is necessary to facilitate HAT at the C3 position. Interestingly, **TS-1a**, contrary to **TS-1b,** is stabilised by hydrogen bonds between the ligands of the metal cofactor (Asp-136, Fe-bound carboxyl group of succinate) or substrate with nearby polar residues (Asn-157, Gln-131, and Asn-70). This effect, however, does not compensate for weakened van der Waals interactions.

Additional computations employed a cluster model consisting of the first coordination shell of Fe and the substrate. The results obtained with this minimal QM model indicate that the position of the substrate as observed in **TS-1b** results solely from its interactions with the binding pocket of the protein. During optimisation of the cluster variant of **TS-1b,** the substrate migrated from its initial position and formed a nearly linear Fe–O–H angle. The activation energy of such a TS is 12.7 kcal mol^−1^ and it is by 2.7 kcal mol^−1^ higher than the barrier associated with a QM-cluster variant of **TS-1a** (see Fig. S7). Therefore, it is the protein environment, not electronic properties of the reactants, that governs the regioselectivity of HAT, which results in preferential formation of **RI-1b**.

#### Desaturation vs. hydroxylation

After the radical intermediate **RI-1b** is formed, the reaction can follow three scenarios. The C3-bound hydrogen can be abstracted from the intermediate to form a desaturated product **P**_**H**_, which is the experimentally observed product [6]. The radical can also donate an unpaired electron to the Fe(III) site and form a carbocation, which is later deprotonated yielding the same **P**_**H**_. Finally, Fe-bound hydroxide can recombine with the radical resulting in a hydroxylated product **P-b**_**OH**_.

In the optimised structure of **RI-1b,** the distance between the C3-bound hydrogen and the oxygen atom of the Fe(III)–OH moiety is 1.97 Å and the Fe–O–H angle is 130°, whereas C10 atom, that hosts the unpaired electron, is located 3.48 Å away from the oxygen atom. Such a geometry is predisposed to desaturation, yet the hydroxylation cannot be ruled out.

The computed Gibbs free energy barriers indicate that HAT is the most favourable process (see Fig. 4). The transition structure associated with the second HAT (**TS-2b**_**H**_) lies 1.4 kcal mol^−1^ lower than TS for OH rebound (**TS-2b**_**OH**_). Despite numerous attempts (QM/MM calculations, combined also with TD-DFT and calculations done for cluster models with fragmental guess), so far we have not managed to optimise a species with substrate derived carbocation and Fe(II)–OH cofactor.

The recent experimental study of AsqJ desaturation activity showed that the C3-epimer of cyclopeptin is converted to the desaturated product, and thus, the hydroxylated species **P-b**_**OH**_ or a carbocation was suggested as intermediates [8]. Our results show that, for the native substrate, the formation of **P-b**_**OH**_ is less favourable than HAT; however, the barrier for hydroxylation is low enough for hydroxylation pathway to be operational when HAT is unfeasible for geometrical reasons (as in the case of the C3-epimer).

**Fig. 6:**
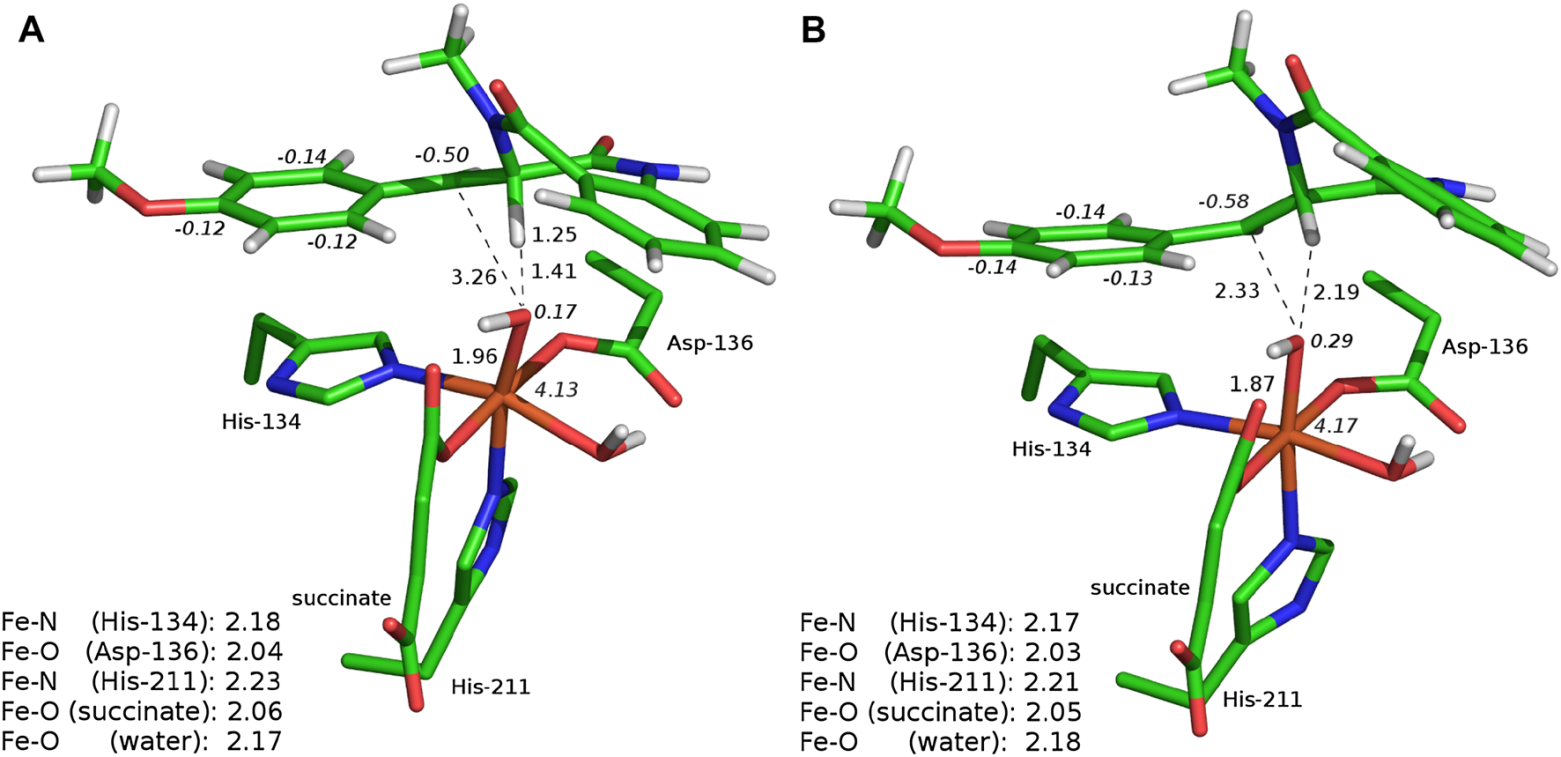
Optimised structures for **TS-2b**_**H**_ (**a**) and **TS-2b**_**OH**_ (**b**). Distances are given in Å and spin populations larger than 0.1 are given in italics

The **TS-2b**_**H**_ and **TS-2b**_**OH**_ are both the early transitions structures. The Fe(III)–OH distance is elongated from 1.86 Å (in **RI-1b**) to 1.96 Å in **TS-2b**_**H**_, whereas it remains almost unchanged (1.87 Å) in **TS-2b**_**OH**_. Spin density population analysis indicates that five unpaired α electrons are located on Fe(III) (spin population of 4.17 and 4.13 for **TS-2b**_**H**_ and **TS-2b**_**OH**_, respectively) and the β electron is delocalised over C10 and the anisyl ring (see Fig. 6).

**Fig. 7:**
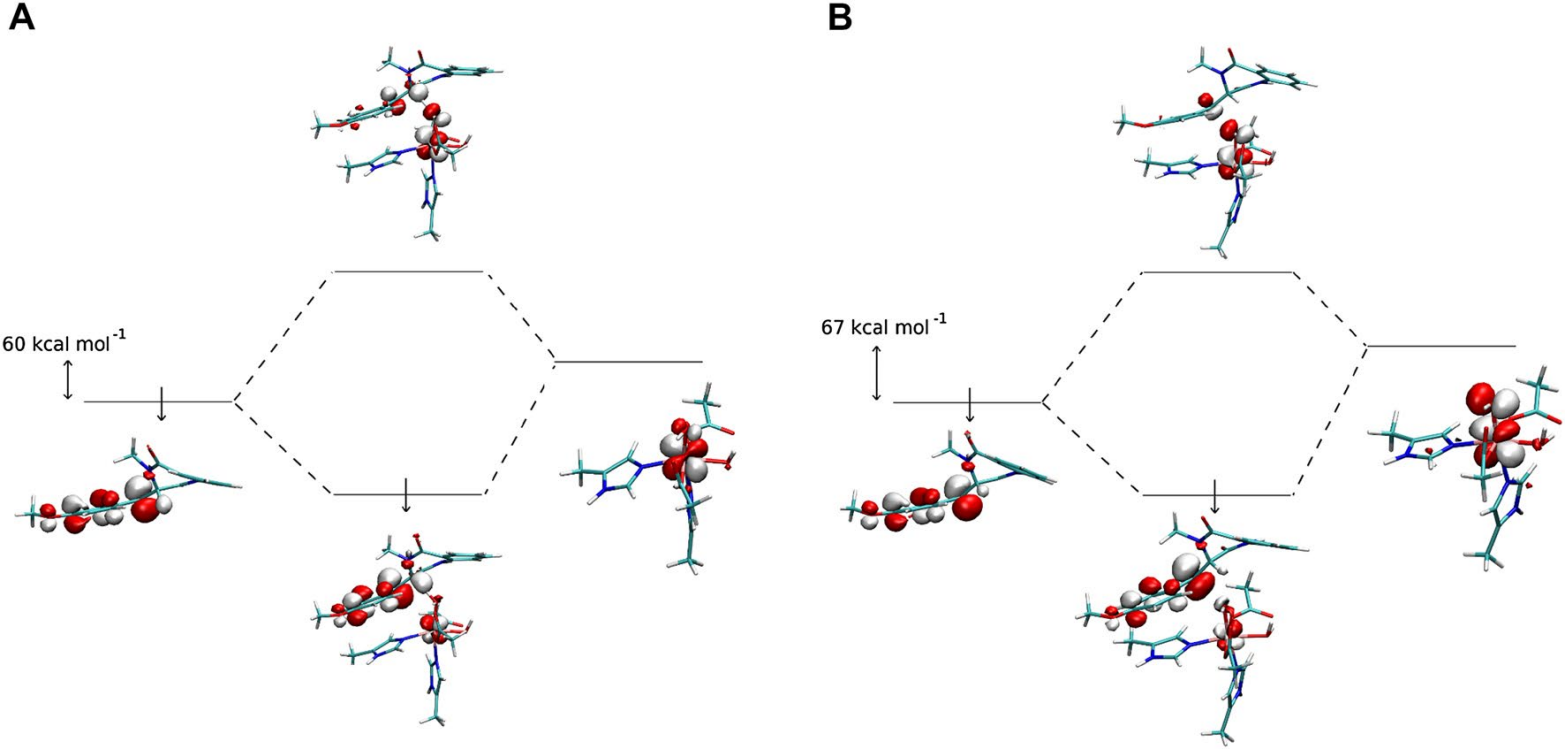
Orbitals mixing in **TS-2b**_**H**_ (**a**) and **TS-2b**_**OH**_ (**b**). Figure rendered using VMD [51]

The preference for desaturation over hydroxylation is most likely rooted in electronic properties of the substrate/metal cofactor pair. The electronic energy barriers calculated for the QM part of the system (ΔE_QM_) show that desaturation is a more feasible process, which is consistent with ONIOM ΔG results. The difference between the two barriers is even larger, it totals to 10.5 kcal mol^−1^ (cf. 1.4 kcal mol^−1^ for ONIOM ΔG). This intrinsic preference for desaturation can be attributed to difference in energy gaps between orbitals that mix in the transition states, i.e., β-dπ*(Fe–OH) (acceptor for an electron in both reactions) and the β-p(C10^·^) for hydroxylation or β-π*(C10–C3) for desaturation, which are the electron source in hydroxylation and desaturation reactions, respectively. To estimate the orbital energy, we performed single-point calculations separately for the Fe(III) site and 4′-methoxycyclopeptin radical, their geometries being the same as in transition structures (**TS-2b**_**H**_ and **TS-2b**_**OH**_) and identified orbitals most similar to ones mixing in the respective transition state structures. As shown in Fig. 7, the energy gap between fragment orbitals that mix in **TS-2b**_**H**_ totals to 60.9 kcal mol^−1^, whereas the gap for **TS-2b**_**OH**_ is by 6.3 kcal mol^−1^ higher. The total fragments’ distortion energies computed for these two TS are 12.0 and 1.5 kcal mol^−1^ for **TS-2b**_**H**_ and **TS-2b**_**OH**_, respectively. The preference for desaturation might also be supported by delocalisation of the radical over the anisyl ring, which usually hinders radical rebound and hence supports entering the alternative reaction channel [11].

**Fig. 8:**
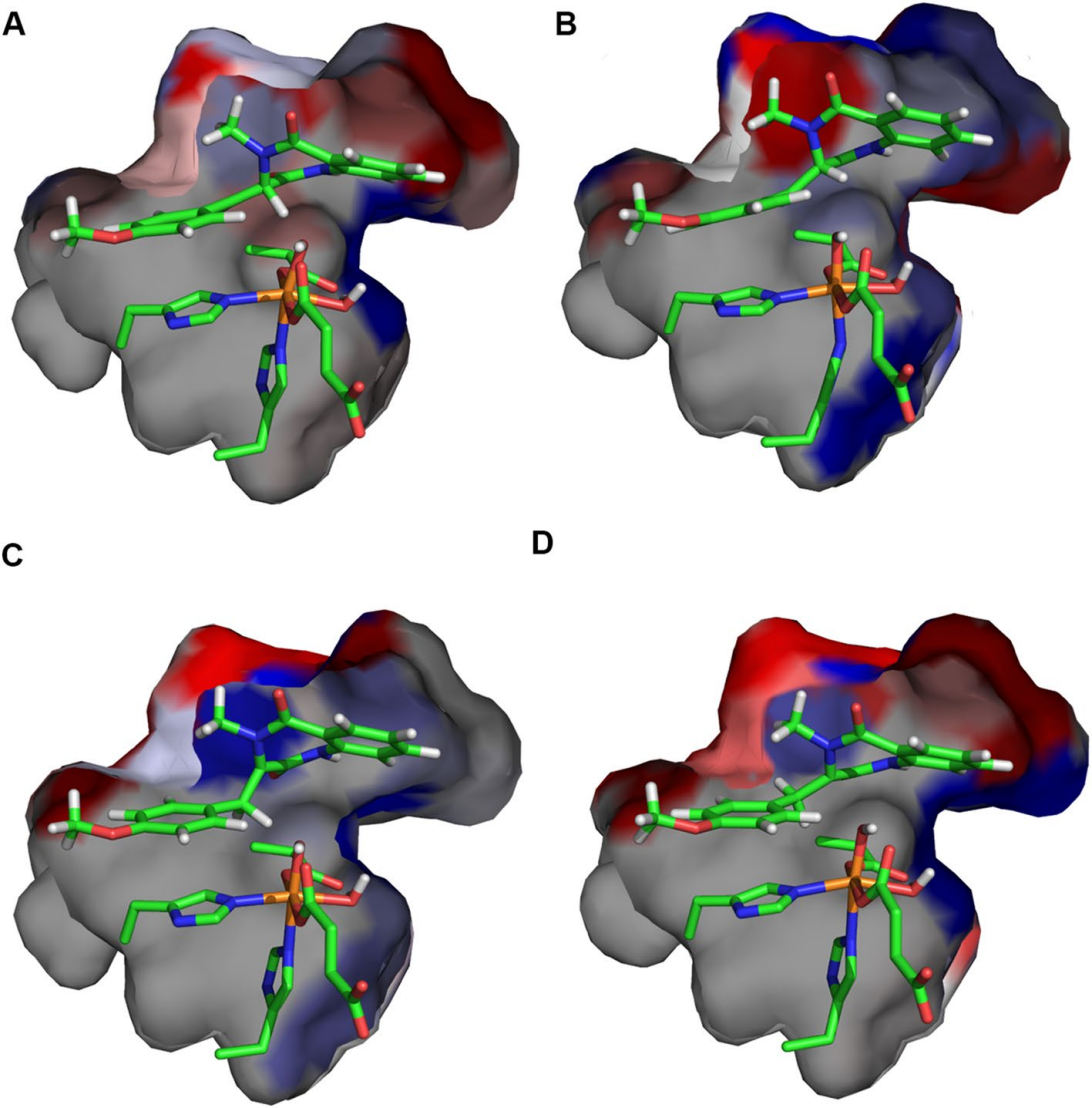
Binding cavity of AsqJ for **TS-2b**_**H**_ (**a**), **TS-2b**_**OH**_ (**b**), **TS-2a**_**H**_ (**c**), and **TS-2a**_**OH**_ (**d**). The amino acids lining the cavity are coloured from blue (stabilising the transition state) to red (destabilising the transition state)

Notably, the energy difference (Δ*G* and Δ*E*) between **TS-2b**_**H**_ and **TS-2b**_**OH**_ decreases when interactions with the protein are taken into account; comparison of Δ*G* or Δ*E* to Δ*E*_QM_ (see Fig. 4) reveals that **TS-2b**_**OH**_ is significantly stabilised by the cavity, whereas **TS-2b**_**H**_ is slightly destabilised. To analyse it further, we performed alanine screening for these two elementary steps. The results (presented in Fig. 8a, b) show that in, **TS-2b**_**H**_, the substrate takes a position that facilitates HAT from the C3 atom at the expense of weakening its interactions with the cavity (Fig. 8a). On the other hand, formation of the bond between C10 and OH in **TS-2b**_**OH**_ requires only very modest shift of the radical intermediate accompanied by stabilising interactions between the bicyclic ring and the cavity (Fig. 8b). Nevertheless, the stabilisation effect of **TS-2b**_**OH**_ does not compensate for the higher electronic energy barrier for hydroxylation. That is why, desaturation is the experimentally observed process. It is possible that replacing some small residues lining the cavity (e.g., Val-72 or Thr-229, see Table S3 and Fig. S8A, B) with more bulky ones might provide additional stabilisation for **TS-2b**_**OH**_ and, consequently, change the reaction selectivity of AsqJ.

The formation of **P**_**H**_, as well as **P-b**_**OH**_, is an exoergic process. The free energy of desaturation is comparable to the free energy of hydroxylation at the C10 position, whereas energies of the QM system indicate that desaturation is more exothermic (see Fig. 4). The thermodynamic cycles for the hydroxylation and desaturation show that the OH rebound is by 4.7 kcal mol^−1^ more favourable than hydrogen atom abstraction at the C3 position by the OH radical. However, this is compensated by the subsequent binding of the water molecule (formed in the desaturation process) to the active site, which results in the stabilisation of the system by 11.4 kcal mol^−1^ (see Figs. S9, S10).

The hydroxylated product **P-b**_**OH**_ adapts such a position within the binding cavity that it is additionally stabilised by the protein environment, which results in comparable free energy of desaturation and hydroxylation.

#### Back to path A: final view on the role of the protein in reaction selectivity

**Fig. 9:**
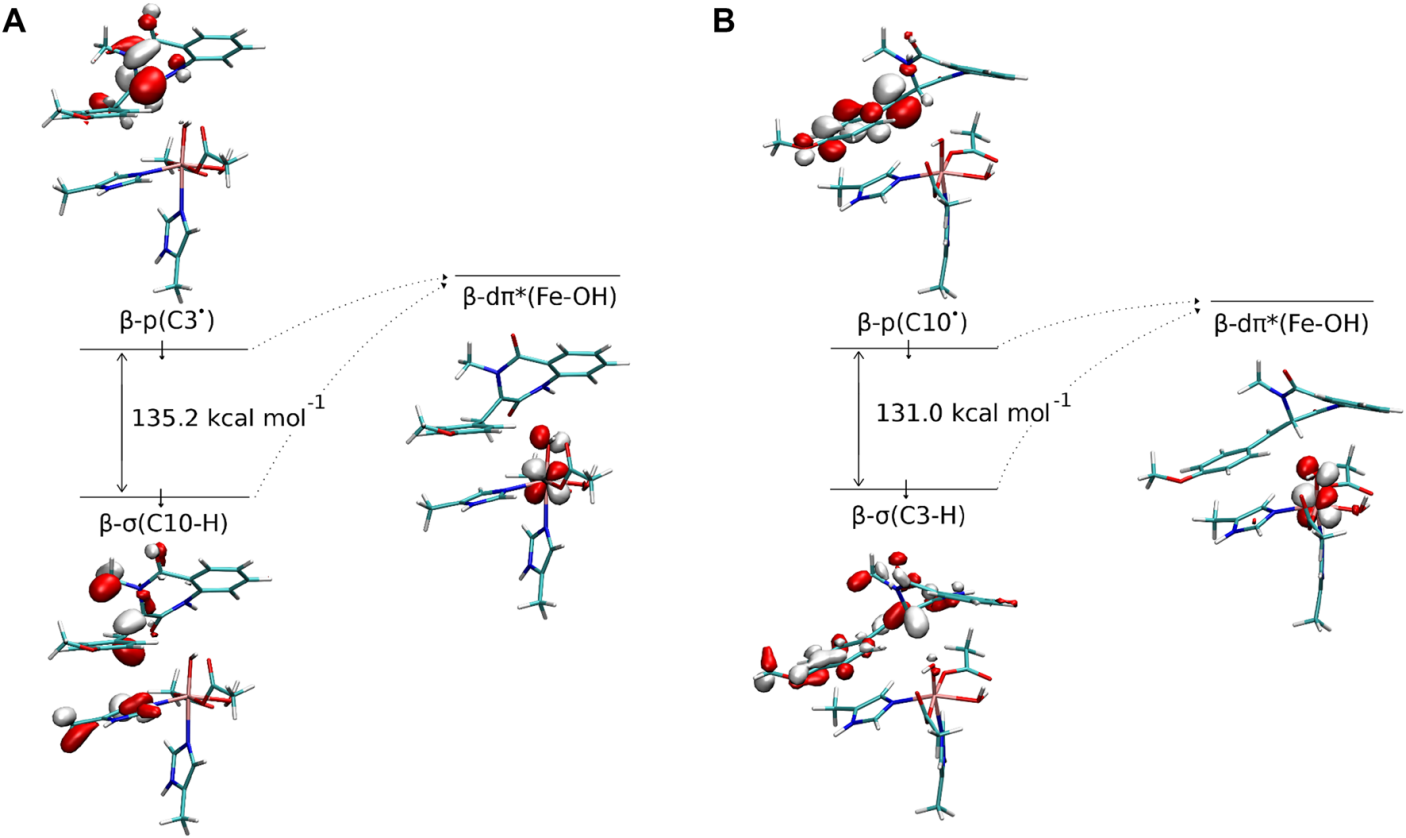
Orbitals in **RI-1a** (**a**) and **RI-1b** (**b**) that donate and accept the electron in desaturation and hydroxylation steps

The less favoured radical intermediate, **RI-1a**, when formed, can also undergo a subsequent hydrogen atom abstraction (proceeding via **TS-2a**_**H**_, shown in Fig. S11A) or hydroxylation (via **TS-2a**_**OH**_, Fig. S11B). The electronic energy barrier ΔE_QM_ for the former is by 3 kcal mol^−1^ higher than for the latter process. The preference for hydroxylation is even more pronounced in the results obtained with the cluster model, where OH rebound occurs without a barrier (Fig. S7). Analysis of the energy gap between orbitals that mix in the transition structure [β-dπ*(Fe–OH) and the β-p(C3^·^) for hydroxylation or β-π*(C3–C10) for desaturation] shows that the gap between orbitals mixing in **TS-2a**_**H**_ totals to 70.2 kcal mol^−1^, and for **TS-2a**_**OH,**_ the gap is only by 3 kcal mol^−1^ higher (see Fig. S12), which indicates a smaller preference towards desaturation than in the **TS-2b**_**H**_/**TS-2b**_**OH**_ pair (6 kcal mol^−1^). The total distortion energy for **TS-2a**_**H**_ is 10.4 kcal mol^−1^, which is slightly lower than the one calculated for **TS-2b**_**H**_ (12.0 kcal mol^−1^), whereas the total distortion energy for **TS-2a**_**OH**_ is larger than for **TS-2b**_**OH**_, and it totals to 4.8 kcal mol^−1^ (cf. 1.5 kcal mol^−1^ for **TS-2b**_**OH**_, shown in Table 1). Moreover, in the electronic structure of **RI-1a** (calculated for radical together with the active site), the energy gap between the β-p(C3^·^) and the lower in energy β-σ(C10–H) totals to 135.2 kcal mol^−1^, which is a larger value than the one calculated for β-p(C10^·^) and β-σ(C3–H) of **RI-1b** (131.0 kcal mol^−1^, as shown in Fig. 9). Therefore, the inherent preference for OH rebound after formation of **RI-1a** can also stem from the increased stability of the σ orbital with respect to the β-p(C3^·^).

**Table 1:** Values of distortion energy obtained for Fe(III) site and 4′-methoxycyclopeptin radical in the same geometries as in transition structures associated with desaturation/hydroxylation

	Path A (kcal mol^−1^)	Path B (kcal mol^−1^)
Desaturation (**TS-2a/b**_**H**_)	10.4	12.0
Hydroxylation (**TS-2a/b**_**OH**_)	4.8 kcal	1.5

Interactions with the binding cavity of the protein change the selectivity of the reaction via destabilisation of **TS-2a**_**OH**_. The results of alanine screening (**TS-2a**_**OH**_ vs **RI-1a**) show again that shortening the distance between the C3 atom and the iron site results in such a re-positioning of the intermediate that its favourable van der Waals interactions with the binding cavity are weakened (see Fig. 8c, d).

This observation together with analysis presented above for the pathway B leads to a general conclusion that due to a slight change in the position of a substrate/radical intermediate transition structures for breaking the C3-H bond (**TS-1a** and **TS-2b**_**H**_) or formation of the C3-O bond (**TS-2a**_**OH**_) feature weaker contacts with the amino acid residues lining the cavity than the preceding intermediates, which results in increase of the free energy barriers.

The desaturated intermediate **P**_**H**_ undergoes a subsequent (AsqJ-catalysed) epoxidation. The process was investigated by QM/MM calculations and the proposed mechanism involves the formation of a bond between C10 and the oxo ligand of the oxoferryl species that yields a C3-centered radical [10]. Comparison of these results with the ones reported here for desaturation shows that both AsqJ-catalysed reactions are initiated at the C10 position. In case of epoxidation, the reported QM/MM barrier for attack at C10 is slightly lower that the one calculated at the QM level, which indicates an additional stabilisation of the transition structure by the binding cavity, similar to **TS-1b** and **TS-2b**_**OH**_ reported here.

### Nonenzymatic rearrangement

**Fig. 10:**
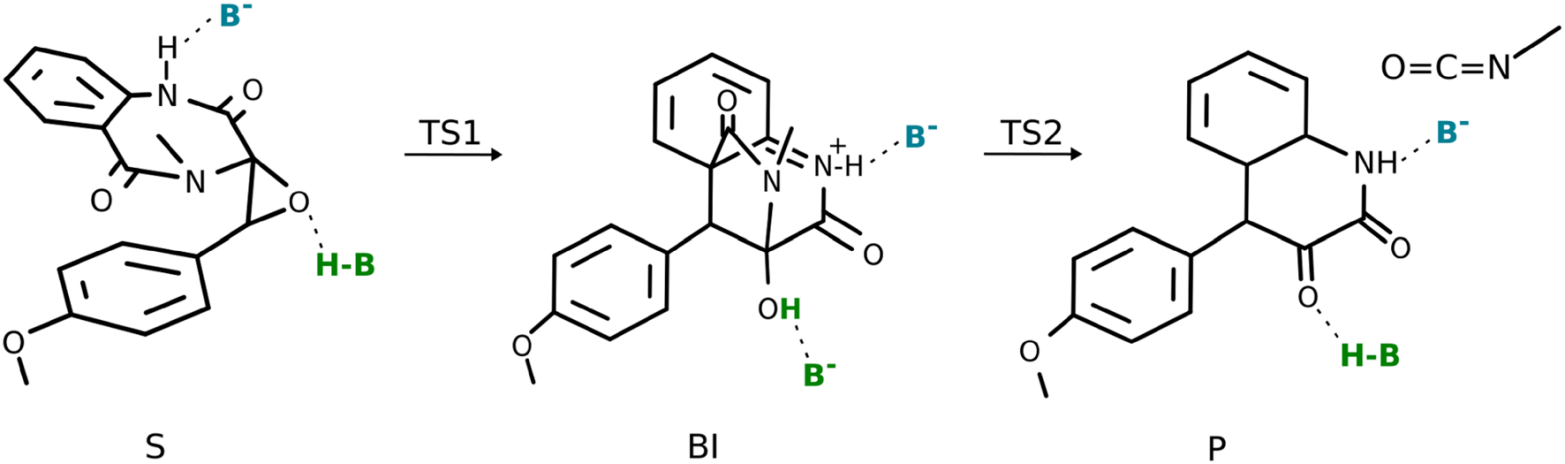
Reaction scheme for the nonenzymatic rearrangement

The final stage of the reaction is a nonenzymatic rearrangement that is supposed to take place in the solvent outside of the enzyme active site. It is a two-step process, which, via elimination of methyl isocyanate, results in formation of the keto form of 4′-methoxyviridicatin (Fig. 10).

The reaction starts with formation of a bridged tricyclic intermediate (**BI**). In water modelled with polarizable continuum model, this step requires crossing a high barrier of 30.5 kcal mol^−1^. The barrier is lowered by 5 kcal mol^−1^ in the presence of two water molecules forming hydrogen bonds with oxirane fragment and the NH group of the benzodiazepinedione moiety (see Fig. S13).

**Fig. 11:**
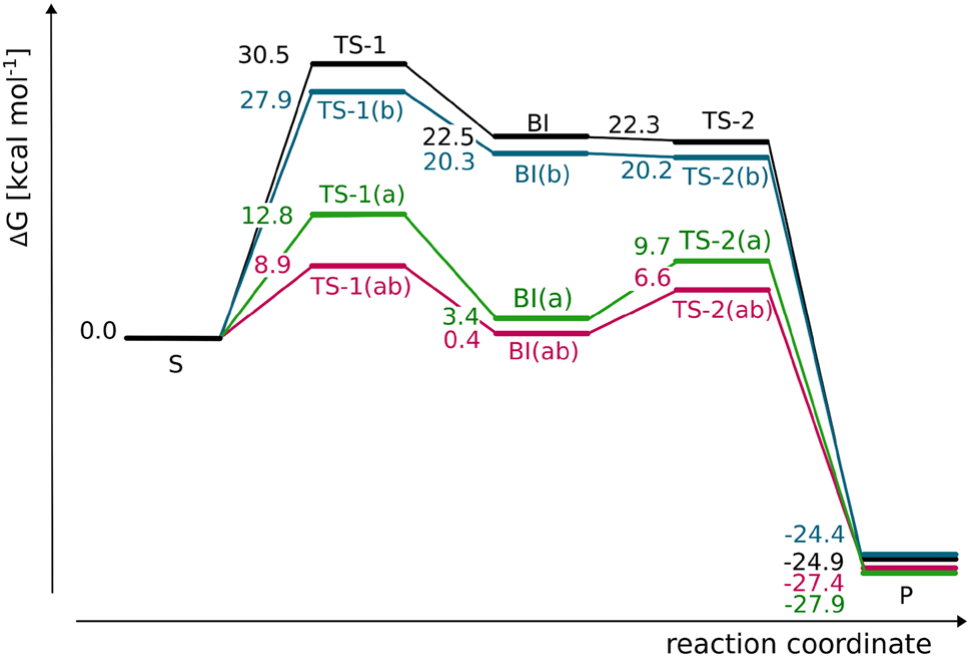
Reaction profiles for rearrangement in presence of implicit water (black line), ascorbate as B^−^ (blue), ascorbic acid as H–B (green), and both ascorbate and ascorbic acid (magenta)

The effect of acid/base catalysis for the rearrangement was investigated with the use of models consisting of the epoxide and acetic acid/acetate or ascorbic acid/ascorbate serving as acid H–B and base B^−^. The general observation is that, to a very good extent, the barrier lowering effects are additive, and the acid molecule, which donates a proton to the epoxide oxygen, is responsible for most of the catalytic effect (see Figs. 11, S14). The ascorbic acid lowers the barrier by 17.7 kcal mol^−1^, whereas the acetic acid by 8.8 kcal mol^−1^. This difference can be partly attributed to larger pK_a_ of acetic acid (4.7 as compared to 4.2 for ascorbic acid). Visual inspection of the imaginary frequency normal mode shows that, along with the C–C bond formation, proton migrates from the donor to the epoxide oxygen, thus forming a hydroxyl group.

Regardless of the presence of base molecule, that can accept proton from the NH group, the N–H bond remains intact, and thus, the resulting bridged bicyclic intermediate has a cationic character. However, the presence of a negatively charged entity interacting with the cationic moiety lowers the Gibbs free energy of the **BI** intermediate by ca. 2 (ascorbate) − 5 (acetate) kcal mol^−1^.

The final step of the reaction, elimination of methyl isocyanate, in the absence of acid molecule occurs without any energy barrier as it leads to the formation of energetically favourable C10 keto group. In systems involving ascorbic acid, the elimination goes over a barrier of ca. 6 kcal mol^−1^ and imaginary frequency associated with this transition state reveals that the proton is transferred back to the ascorbate anion.

## Conclusions

The proposed mechanism for AsqJ-catalysed desaturation involves HAT at the C10 position followed by second hydrogen abstraction from the neighbouring C3 atom. The regioselectivity of the first HAT stems from favourable interactions with the hydrophobic residues lining the binding cavity, whereas product selectivity is dictated by electronic properties of the reactants. However, the electronic preference for desaturation is partially reduced by stabilising effect of the binding pocket that lowers the barrier for the hydroxylation reaction. This observation opens up new possibilities for switching reaction selectivity of AsqJ by introducing mutations within the binding pocket.

## Electronic supplementary material

Below is the link to the electronic supplementary material.

RMSD plots for MD simulations (**Fig. S1** and **S2**), crucial distances and angles obtained during model preparation (Table S1), the structures of the active site of AsqJ obtained during model preparation (**Fig. S3**), the partially occupied natural orbitals for spin densities for **TS-1a** and **TS-1b** (**Fig. S4**), the ONIOM energy barriers calculated for mutated variants of **TS-1** and **TS-1b** (Table S2), **TS-2b**_**H**_ and **TS-2b**_**OH**_ (Table S3), **TS-2a**_**H**_ and **TS-2a**_**OH**_ (Table S4), compared optimised structures of **TS-1a** and **TS-1b** (**Fig. S5**), the binding site of AsqJ in stick representation coloured according to TS stabilisation/destabilisation for **TS-1a** and **TS-1b** (**Fig. S6**), **TS-2b**_**H**_, **TS-2b**_**OH**_, **TS-2a**_**H**_, and **TS-2a**_**OH**_ (**Fig. S8**), the reaction profile calculated for a cluster model of AsqJ (**Fig. S7**), the thermodynamic cycles for hydroxylation and desaturation in path B (**Fig. S9**) and A (**Fig. S10**), optimised structures for **TS-2a**_**H**_ and **TS-2a**_**OH**_ (**Fig. S11**), orbitals mixing in **TS-2b**_**H**_ and **TS-2b**_**OH**_ (**Fig. S12**), reaction profiles for rearrangement and elimination in different systems (**Fig. S13** and **S14**), and absolute and relative energies of stationary points (Tables S5-S10). Cartesian coordinates of stationary points are available on ioChem-BD:  https://iochem.udg.edu:8443/browse/handle/100/435 (PDF 3212 kb)

## Abbreviations

2OG: 2-oxoglutarate
HAT: Hydrogen atom transfer
MD: Molecular dynamics
ODD: 2-oxoglutarate-dependent dioxygenases
QM/MM: Quantum mechanics/molecular mechanics
TauD: Taurine/α-ketoglutarate dioxygenase
