# Supplementary material for: On how the binding cavity of AsqJ dioxygenase controls the desaturation reaction regioselectivity: a QM/MM study
Source: J Biol Inorg Chem. 2018 Jun 6;23(5):795–808. doi: 10.1007/s00775-018-1575-3 (PMC6015105; doi:10.1007/s00775-018-1575-3)
Supplement: Supplementary file 1 — RMSD plots for MD simulations (Fig. S1 and S2), crucial distances and angles obtained during model preparation (Table S1), the structures of the active site of AsqJ obtained during model preparation (Fig. S3), the partially occupied natural orbitals for spin densities for TS-1a and TS-1b (Fig. S4), the ONIOM energy barriers calculated for mutated variants of TS-1 and TS-1b (Table S2), TS-2bH and TS-2bOH (Table S3), TS-2aH and TS-2aOH (Table S4), compared optimised structures of TS-1a and TS-1b (Fig. S5), the binding site of AsqJ in stick representation coloured according to TS stabilisation/destabilisation for TS-1a and TS-1b (Fig. S6), TS-2bH, TS-2bOH, TS-2aH, and TS-2aOH (Fig. S8), the reaction profile calculated for a cluster model of AsqJ (Fig. S7), the thermodynamic cycles for hydroxylation and desaturation in path B (Fig. S9) and A (Fig. S10), optimised structures for TS-2aH and TS-2aOH (Fig. S11), orbitals mixing in TS-2bH and TS-2bOH (Fig. S12), reaction profiles for rearrangement and elimination in different systems (Fig. S13 and S14), and absolute and relative energies of stationary points (Tables S5-S10). Cartesian coordinates of stationary points are available on ioChem-BD: https://iochem.udg.edu:8443/browse/handle/100/435 (PDF 3212 kb) [file 775_2018_1575_MOESM1_ESM.pdf]

Supporting Information

On how the binding cavity of AsqJ dioxygenase controls the desaturation reaction regioselectivity: a QM/MM study

Zuzanna Wojdyła, Tomasz Borowski

Jerzy Haber Institute of Catalysis and Surface Chemistry, Polish Academy of Sciences, Niezapominajek 8, PL-30239 Krakow, Poland

MD simulations

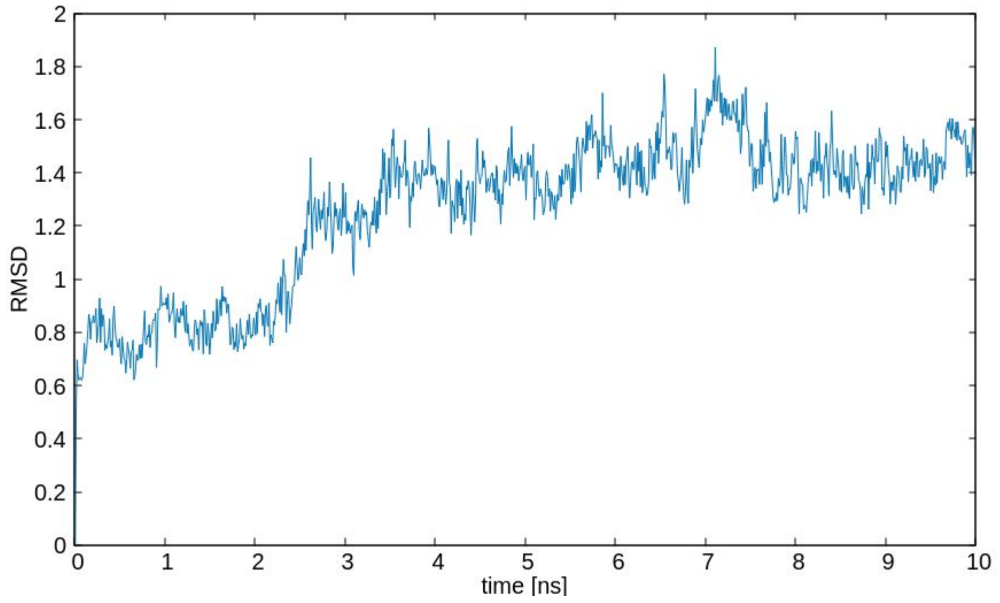

Fig. S1. RMSD vs time plot for AsqJ:2OG:4'-methoxycyclopeptin complex

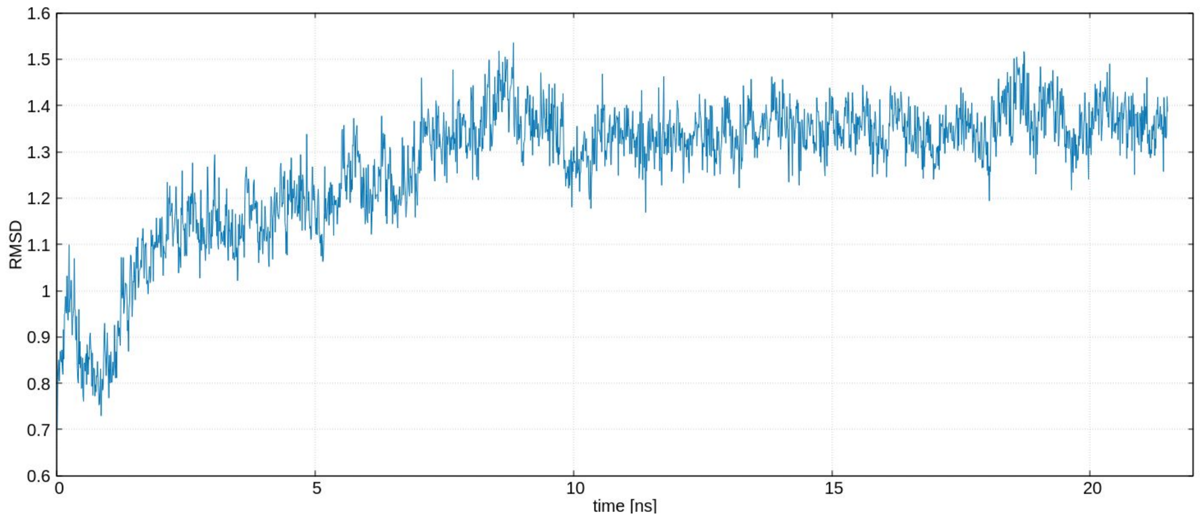

Fig. S2. RMSD vs time plot for AsqJ:succinate:4'-methoxycyclopeptin complex

Table S1. Crucial distances and angles during model preparation. For MD simulations average distances in both subunits are given.

	C3 position		C10 position	
	H-O distance [Å]	H---O-Fe angle [deg]	H-O distance [Å]	H---O-Fe angle [deg]
crystal structure with added H atoms	2.24	113	3.14	77
AsqJ:2OG MD average	2.4±0.1 / 2.8±0.2	111±7/ 123±5	3.9±0.3 / 3.2±0.3	77±3 / 81±4
representative structure from the dominant cluster	2.45	113	4.08	78
AsqJ:2OG ONIOM optimisation	2.43	103	3.38	67
AsqJ:succinate ONIOM optimisation	2.26	148	2.75	92
AsqJ:succinate MD average	2.7±0.1 / 2.8±0.4	157±6/ 148±7	2.7±0.1 / 3.0±0.4	105±5 / 105±6
representative structure from the dominant cluster	2.45	154	2.78	101
relaxed AsqJ:succinate ONIOM optimisation	2.13	146	2.73	90

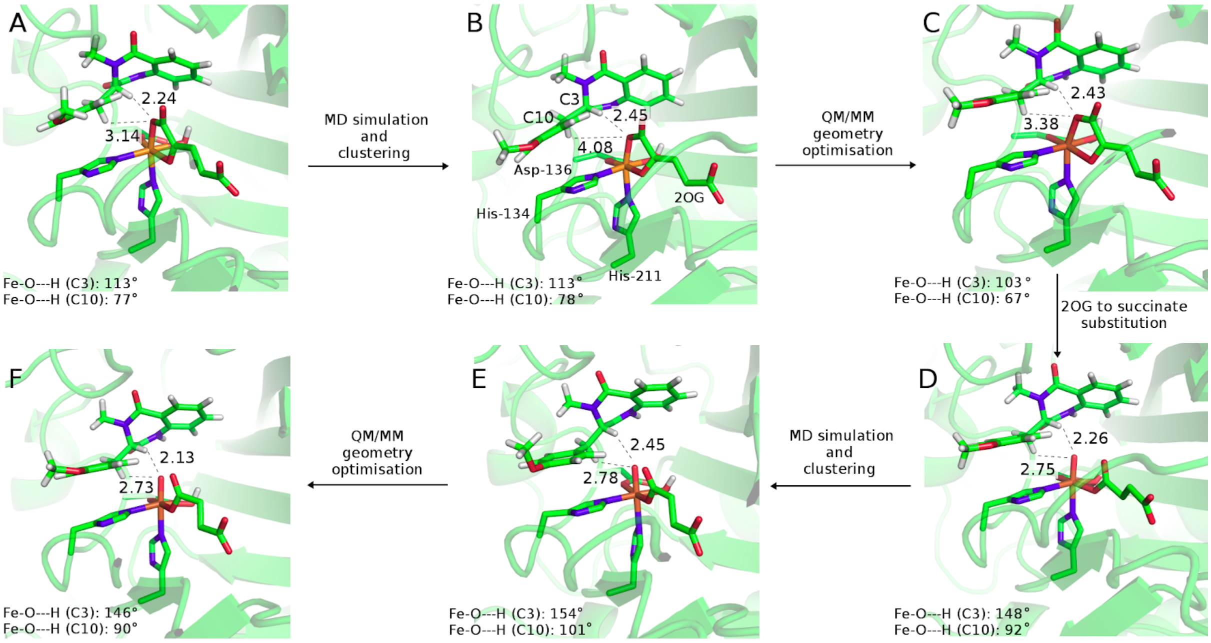

Fig. S3. The crucial H-O distances and Fe-O---H angles for crystal structure with added hydrogen atoms (A), representative structure of the dominant cluster from the AsqJ:2OG complex MD simulation (B), the ONIOM optimised structure of the AsqJ:2OG complex (C), the ONIOM optimised structure of the AsqJ:succinate complex (D), representative structure of the dominant cluster from the AsqJ:succinate complex MD simulation (E), the ONIOM optimised structure of the relaxed AsqJ:succinate complex

ONIOM calculations

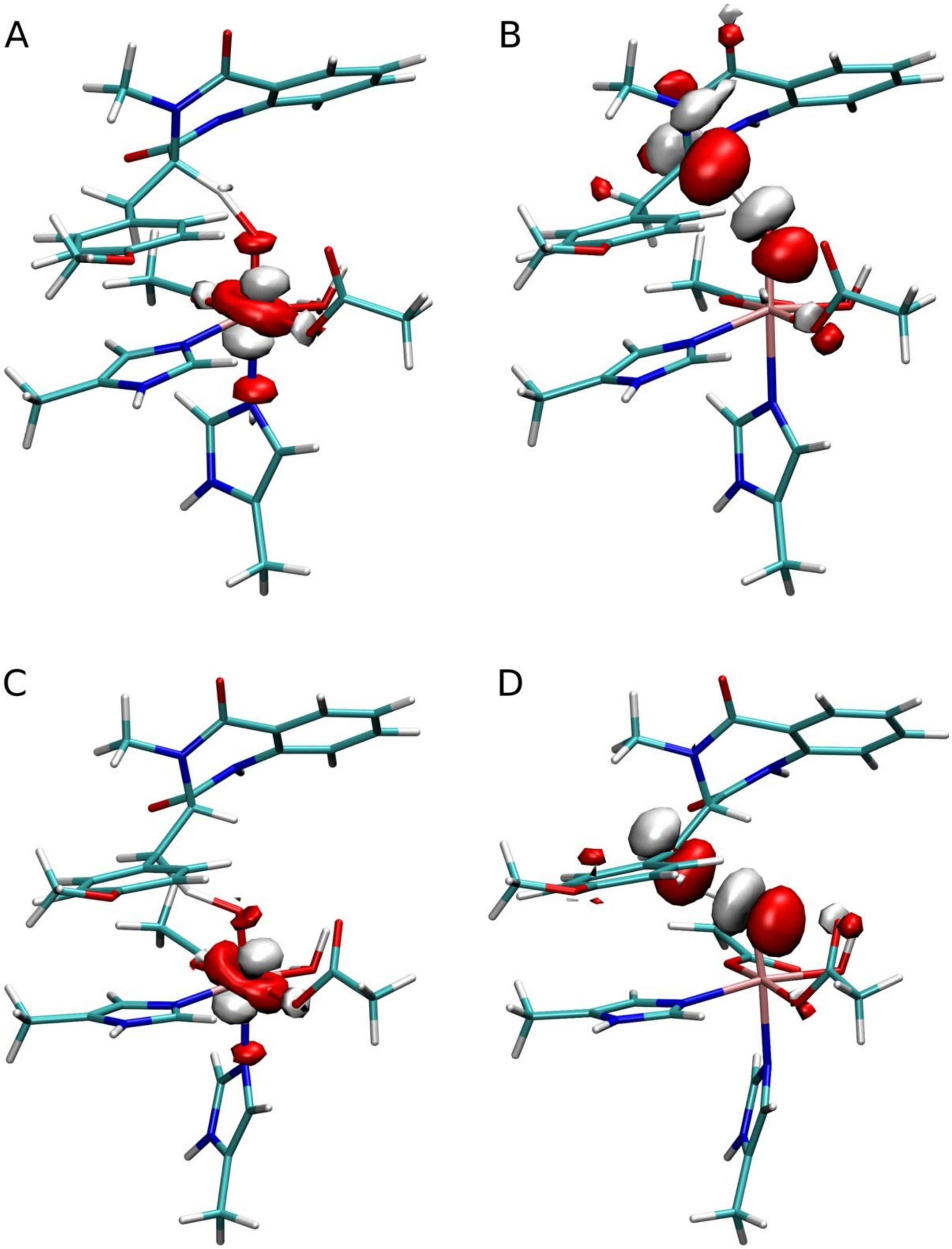

Fig. S4. Contours of the key natural orbitals for spin density: with positive eigenvalue (α-spin) (A) and negative eigenvalue (β-spin) (B) for **TS-1a** and analogous orbitals for for **TS-1b**, (C) and (D), respectively. Occupancy of the orbitals totals to 0.75 and 0.86 for **TS-1a** and **TS-1b**, respectively.

Table S2. The ONIOM energy barriers (ΔE) for mutated variants of **TS-1a** and **TS-1b** and the change in barrier caused by the mutation (ΔΔE). Coulomb, van der Waals and bonded contributions are reported as $\Delta\Delta E_{Coulomb}$, $\Delta\Delta E_{vdW}$ and $\Delta\Delta E_{bonded}$, respectively. Values are given in kcal mol^{-1}.

	**TS-1a**					**TS-1b**				
	ΔE	ΔΔE	$\Delta\Delta E_{Coulomb}$	$\Delta\Delta E_{vdW}$	$\Delta\Delta E_{bonded}$	ΔE	ΔΔE	$\Delta\Delta E_{Coulomb}$	$\Delta\Delta E_{vdW}$	$\Delta\Delta E_{bonded}$
wt	18.12					15.31				
Arg-271	17.22	0.91	1.02	0.00	-0.12	14.79	0.52	0.41	0.11	0.00
Ile-273	18.11	0.02	-0.37	0.36	0.02	15.67	-0.36	-0.11	-0.27	0.03
Asn-70	18.37	-0.25	-0.54	0.28	0.01	15.03	0.29	0.39	-0.17	0.06
Val-72	17.98	0.15	0.38	-0.18	-0.07	15.59	-0.28	-0.3	-0.04	0.06
Leu-73	18.10	0.02	0.00	0.03	-0.02	15.14	0.18	0.02	0.15	0.01
Leu-79	18.12	0.01	0.00	0.01	0	15.35	-0.04	-0.02	-0.01	-0.01
Met-118	17.69	0.43	0.33	0.09	0.01	15.88	-0.57	-0.75	0.15	0.04
Met-122	18.00	0.12	0.14	-0.05	0.02	15.5	-0.18	-0.07	-0.11	0.00
Gln-131	19.04	-0.92	-0.45	-0.41	-0.07	14.26	1.06	0.23	0.61	0.21
Met-137	18.08	0.04	0.04	-0.06	0.05	15.39	-0.08	-0.09	-0.05	0.06
Phe-139	18.12	0.01	-0.09	0.07	0.02	15.37	-0.05	0.07	-0.11	-0.02
Ser-140	17.95	0.17	0.18	-0.02	0.00	15.24	0.07	0.34	-0.26	-0.01
Asn-157	18.28	-0.16	0.17	-0.3	-0.03	14.91	0.41	0.51	-0.03	-0.07
Leu-159	17.83	0.30	0.00	0.29	-0.01	15.24	0.07	-0.11	0.19	-0.02
Ile-205	17.57	0.55	0.47	0.05	0.03	15.9	-0.59	0.00	-0.72	0.12
Thr-227	18.03	0.09	-0.02	0.09	0.01	14.82	0.49	-0.13	0.66	-0.04
Thr-229	17.77	0.35	-0.26	0.61	0.00	15.95	-0.64	-0.34	-0.25	-0.05
Leu-238	17.88	0.25	0.01	0.22	0.01	15.45	-0.14	-0.01	-0.12	-0.02
water-1	19.24	-1.11	-2.22	1.03	0.06	16.01	-0.7	-4.22	3.31	0.2
water-2	16.35	1.77	-0.31	1.91	0.17	16.83	-1.51	-1.27	-0.28	0.04
**Sum over AA**		**2.09**	**1.01**	**1.08**	**-0.14**		**0.16**	**0.04**	**-0.25**	**0.35**
Sum over waters		0.66	-2.53	2.94	0.23		-2.21	-5.94	3.03	0.24
TOTAL		2.75	-1.52	4.02	0.09		-2.05	-5.45	2.78	0.59

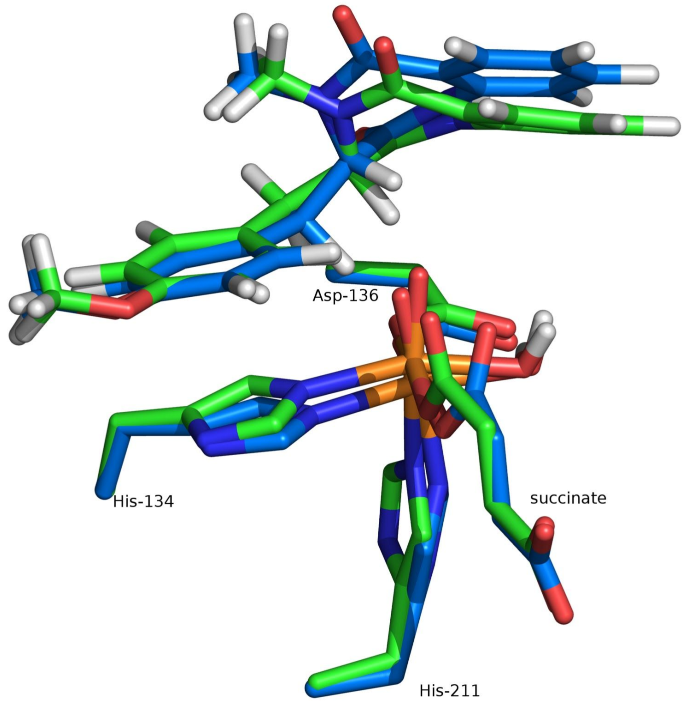

Fig. S5. Compared optimised structures of **TS-1a** (green) and **TS-1b** (blue)

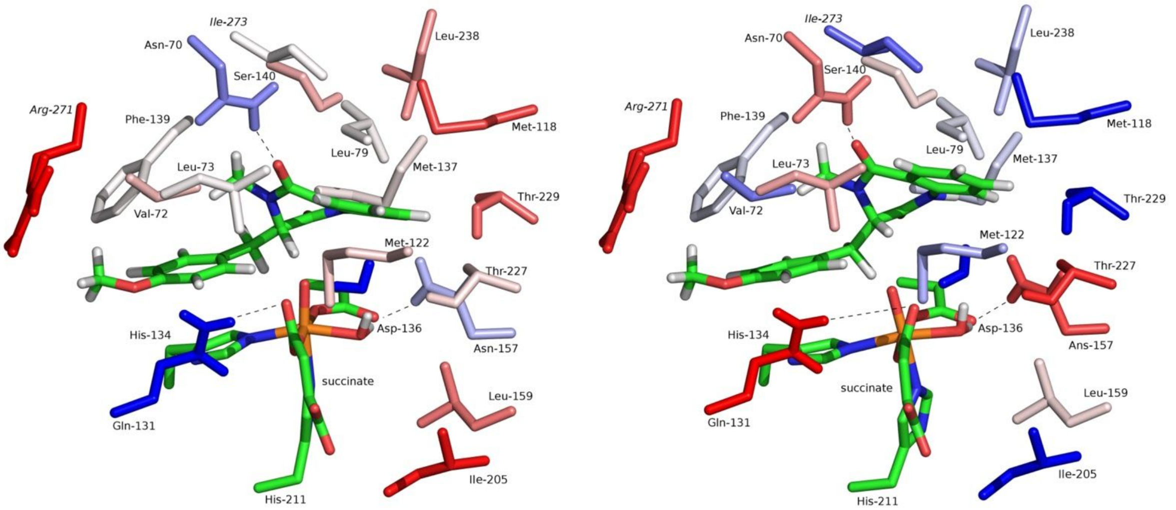

Fig. S6. The amino acid residues lining the binding cavity of AsqJ in **TS-1a** (A) and **TS-1b** (B) colored from blue (stabilising the transition state) to red (destabilising the transition state). The residues labelled in italics come from the second subunit.

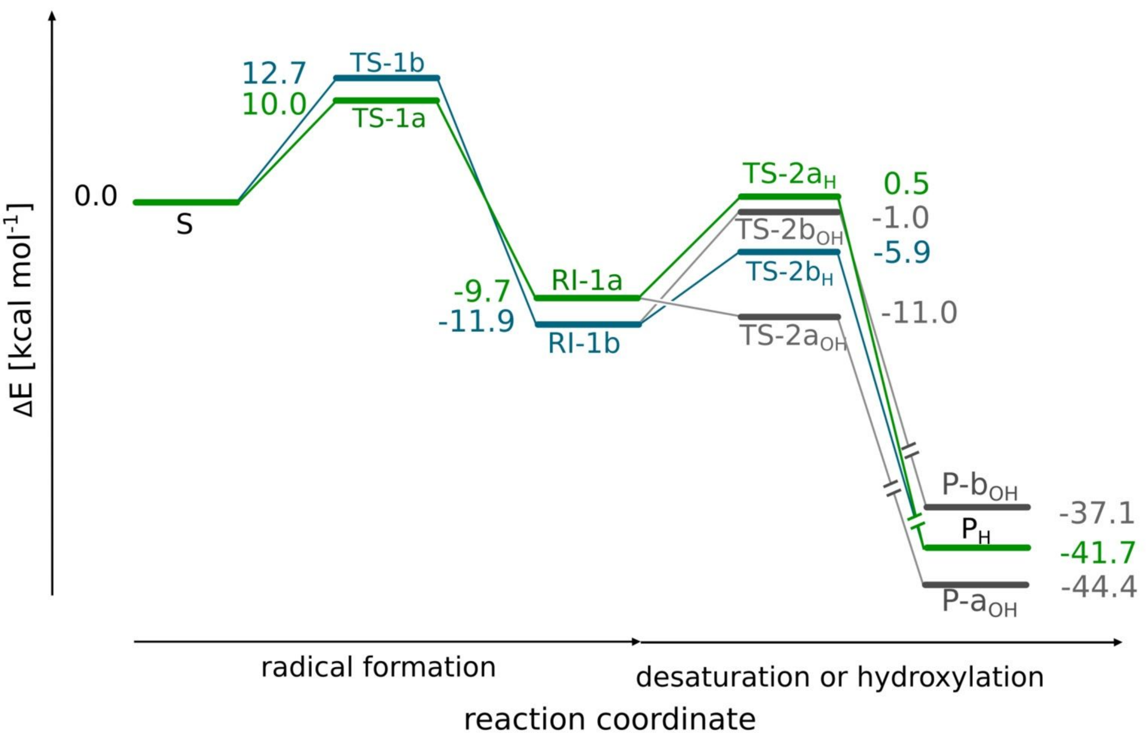

Fig. S7. Reaction energy profile for the cluster model.

Table S3. The ONIOM energy barriers (ΔE) for mutated variants of **TS-2b$_H$** and **TS-2b$_{OH}$** and changes in barriers caused by the mutation (ΔΔE). Coulomb, van der Waals and bonded contributions are reported as $\Delta\Delta E_{Coulomb}$, $\Delta\Delta E_{vdW}$ and $\Delta\Delta E_{bonded}$, respectively. Values are given in kcal mol^{-1}.

	**TS-2b$_H$**					**TS-2b$_{OH}$**				
	ΔE	ΔΔE	$\Delta\Delta E_{Coulomb}$	$\Delta\Delta E_{vdW}$	$\Delta\Delta E_{bonded}$	ΔE	ΔΔE	$\Delta\Delta E_{Coulomb}$	$\Delta\Delta E_{vdW}$	$\Delta\Delta E_{bonded}$
wt	6.60					7.81				
Arg-271	6.84	-0.24	-0.11	0.05	-0.18	9.04	-1.23	-1.21	0.04	-0.05
Ile-273	6.71	-0.10	-0.42	0.32	0.00	8.50	-0.68	-0.60	-0.16	0.07
Asn-70	6.66	-0.06	-0.40	0.30	0.04	7.26	0.55	0.46	0.06	0.03
Val-72	6.46	0.15	0.55	-0.37	-0.03	7.81	0.00	0.24	-0.23	-0.02
Leu-73	6.65	-0.05	-0.02	-0.02	-0.02	7.77	0.04	0.01	0.03	0.00
Leu-79	6.60	0.00	0.00	0.02	-0.01	7.87	-0.06	-0.01	-0.04	-0.01
Met-118	5.96	0.65	0.51	0.14	-0.01	8.11	-0.30	-0.50	0.18	0.03
Met-122	6.56	0.05	0.01	0.07	-0.03	8.04	-0.23	-0.20	0.00	-0.03
Gln-131	6.77	-0.16	0.09	-0.21	-0.05	7.77	0.04	-0.90	0.78	0.15
Met-137	6.42	0.18	0.19	-0.03	0.02	7.97	-0.16	-0.20	-0.04	0.07
Phe-139	6.44	0.16	-0.06	0.16	0.07	7.45	0.36	0.24	0.15	-0.03
Ser-140	6.09	0.52	0.58	-0.06	0.00	7.26	0.55	0.95	-0.38	-0.03
Asn-157	7.20	-0.60	-0.32	-0.28	-0.01	8.13	-0.31	-0.37	0.14	-0.08
Leu-159	6.55	0.05	0.01	0.07	-0.02	7.92	-0.10	-0.07	-0.03	-0.01
Ile-205	6.57	0.04	0.02	0.00	0.02	8.57	-0.76	-0.06	-0.83	0.13
Thr-227	6.47	0.13	-0.02	0.11	0.05	7.33	0.48	0.38	0.13	-0.03
Thr-229	5.93	0.67	0.17	0.54	-0.03	8.12	-0.31	-0.26	0.02	-0.08
Leu-238	6.33	0.28	0.02	0.26	0.00	7.93	-0.12	0.02	-0.14	0.00
water-1	7.72	-1.12	-1.67	0.50	0.06	5.57	2.24	3.24	-0.84	-0.17
water-2	5.40	1.21	-0.65	1.67	0.18	7.22	0.59	-0.11	0.70	-0.01
**Sum over AA**		**1.67**	0.81	1.06	-0.20		**-2.25**	-2.06	-0.31	0.12
Sum over waters		0.09	-2.32	2.17	0.23		2.83	3.14	-0.13	-0.18
TOTAL		**1.75**	-1.51	3.23	0.03		**0.58**	1.07	-0.44	-0.06

Table S4. The ONIOM energy barriers (ΔE) for mutated variants of **TS-2a$_H$** and **TS-2a$_{OH}$** and the change in barrier caused by the mutation (ΔΔE). Coulomb, van der Waals and bonded contributions are reported as $\Delta\Delta E_{Coulomb}$, $\Delta\Delta E_{vdW}$ and $\Delta\Delta E_{bonded}$, respectively. Values are given in kcal mol^{-1}.

	**TS-2a$_H$**					**TS-2a$_{OH}$**				
	ΔE	ΔΔE	$\Delta\Delta E_{Coulomb}$	$\Delta\Delta E_{vdW}$	$\Delta\Delta E_{bonded}$	ΔE	ΔΔE	$\Delta\Delta E_{Coulomb}$	$\Delta\Delta E_{vdW}$	$\Delta\Delta E_{bonded}$
wt	6.11					3.37				
Arg-271	5.87	0.24	0.26	0.08	-0.09	2.45	0.92	1.13	-0.09	-0.12
Ile-273	5.51	0.60	0.27	0.31	0.03	2.61	0.76	-0.09	0.84	0.01
Asn-70	8.10	-1.99	-1.84	-0.04	-0.11	4.72	-1.35	-1.74	0.47	-0.08
Val-72	6.19	-0.08	-0.29	0.26	-0.04	3.03	0.34	0.27	0.18	-0.12
Leu-73	5.78	0.32	0.00	0.31	0.01	3.31	0.06	-0.03	0.10	-0.02
Leu-79	6.06	0.05	0.00	0.05	0.00	3.36	0.01	0.00	0.01	0.00
Met-118	6.11	0.00	0.13	-0.11	-0.01	2.63	0.74	0.80	-0.01	-0.05
Met-122	5.96	0.15	-0.03	0.14	0.03	3.30	0.06	0.01	0.14	-0.09
Gln-131	6.14	-0.03	0.93	-0.79	-0.16	2.12	1.25	0.15	1.00	0.09
Met-137	6.19	-0.09	0.02	-0.10	-0.01	3.36	0.00	0.11	-0.12	0.01
Phe-139	5.30	0.81	0.37	0.38	0.06	2.79	0.57	0.24	0.28	0.05
Ser-140	6.08	0.03	-0.33	0.34	0.03	2.93	0.44	0.12	0.32	0.01
Asn-157	6.29	-0.18	0.15	-0.20	-0.13	4.44	-1.07	-0.42	-0.51	-0.14
Leu-159	6.28	-0.17	-0.01	-0.15	-0.01	3.52	-0.16	0.05	-0.19	-0.02
Ile-205	6.36	-0.26	-0.07	-0.19	0.01	3.35	0.02	0.03	0.00	-0.01
Thr-227	6.14	-0.03	0.31	-0.33	-0.01	4.05	-0.68	-0.61	-0.14	0.07
Thr-229	6.23	-0.12	-0.62	0.48	0.03	2.81	0.56	-0.44	1.03	-0.04
Leu-238	6.35	-0.24	0.02	-0.25	0.00	3.15	0.22	0.04	0.19	0.00
water-1	6.95	-0.84	-0.13	-0.68	-0.02	5.21	-1.85	-0.21	-1.61	-0.02
water-2	4.12	1.98	1.34	0.69	-0.05	-0.55	3.92	1.42	2.38	0.12
**Sum over AA**		**-0.97**	-0.73	0.16	-0.40		**2.69**	-0.37	3.51	-0.45
Sum over waters		1.14	1.21	0.00	-0.07		2.07	1.21	0.77	0.10
TOTAL		**0.17**	0.48	0.16	-0.47		**4.77**	0.84	4.28	-0.35

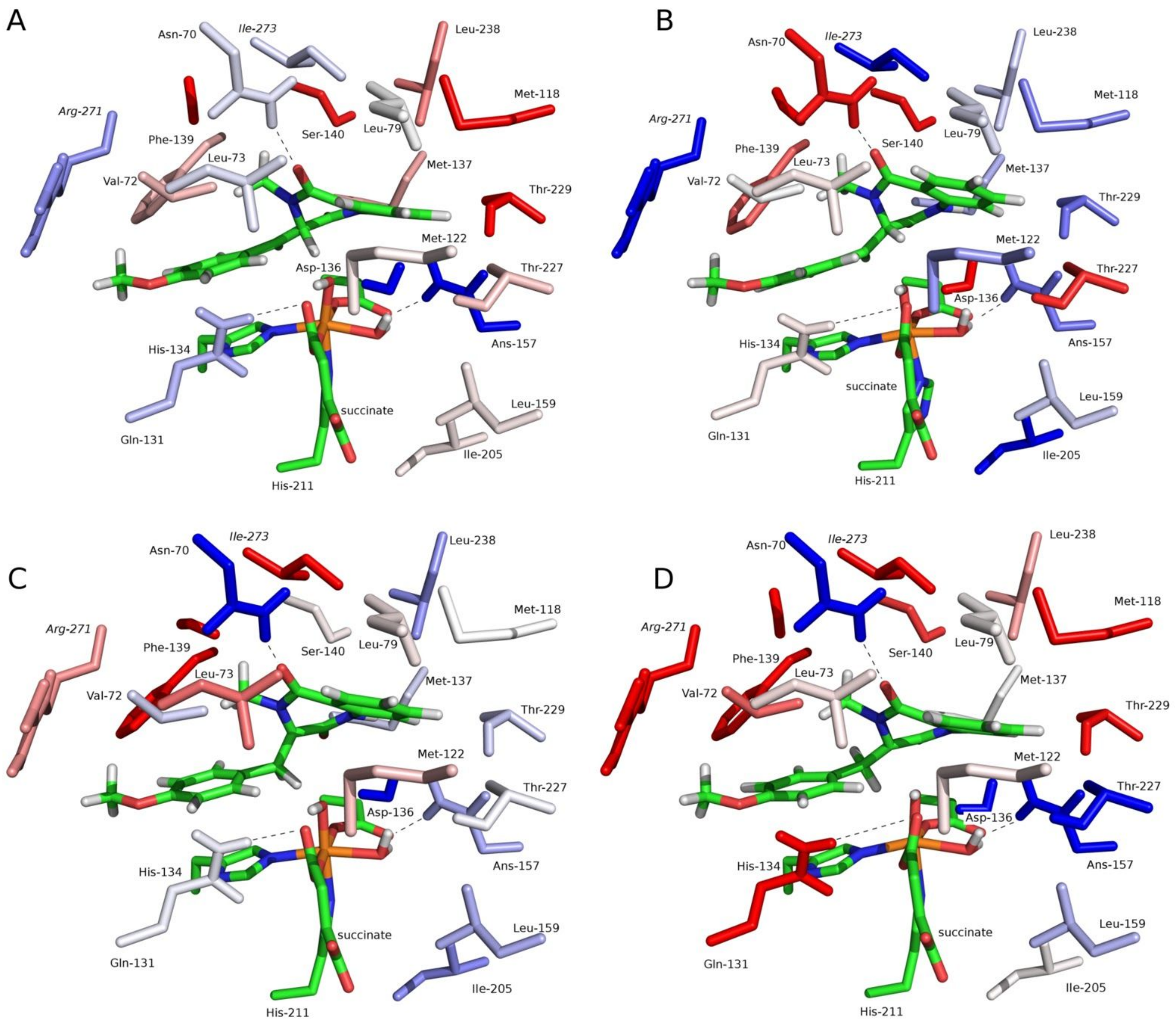

Fig. S8. The amino acid residues lining the binding cavity of AsqJ in **TS-2b$_{\mathbf{H}}$** (A), **TS-2b$_{\mathbf{OH}}$** (B), **TS-2a$_{\mathbf{H}}$** (C), **TS-2a$_{\mathbf{OH}}$** (D) colored from blue (stabilising the transition state) to red (destabilising the transition state). The residues labelled in italics come from the second subunit.

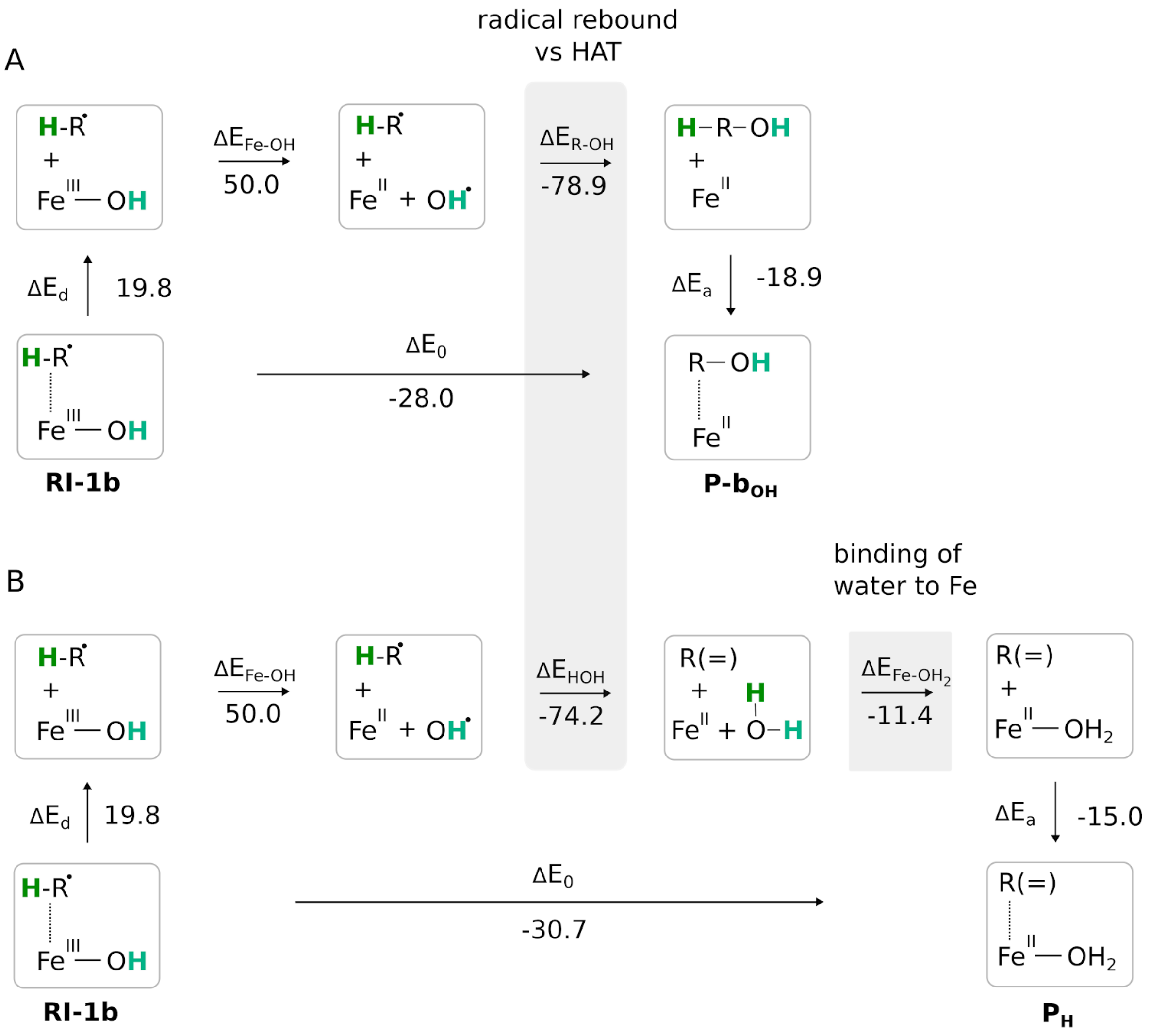

Fig. S9. The thermodynamic cycle for hydroxylation (A) and desaturation (B) in path B. Both processes are initiated by radical $R^{\bullet}$ dissociation followed by homolytic cleavage of the Fe(III)-OH bond. Later, in the hydroxylation process OH radical recombines with $R^{\bullet}$ and the hydroxylated product associates to the Fe(II) site. The desaturation process was divided into HAT from the radical by the OH radical, association of the formed water molecule to the Fe(II) site and, finally, association of the desaturation product to the site. The reaction energies (ΔE_0) are the sum of the energies of the described steps. The values are given in kcal mol^{-1} and were calculated at the B3LYP-D3/def2-TZVP//B3LYP-D3/def2-SVP level of theory.

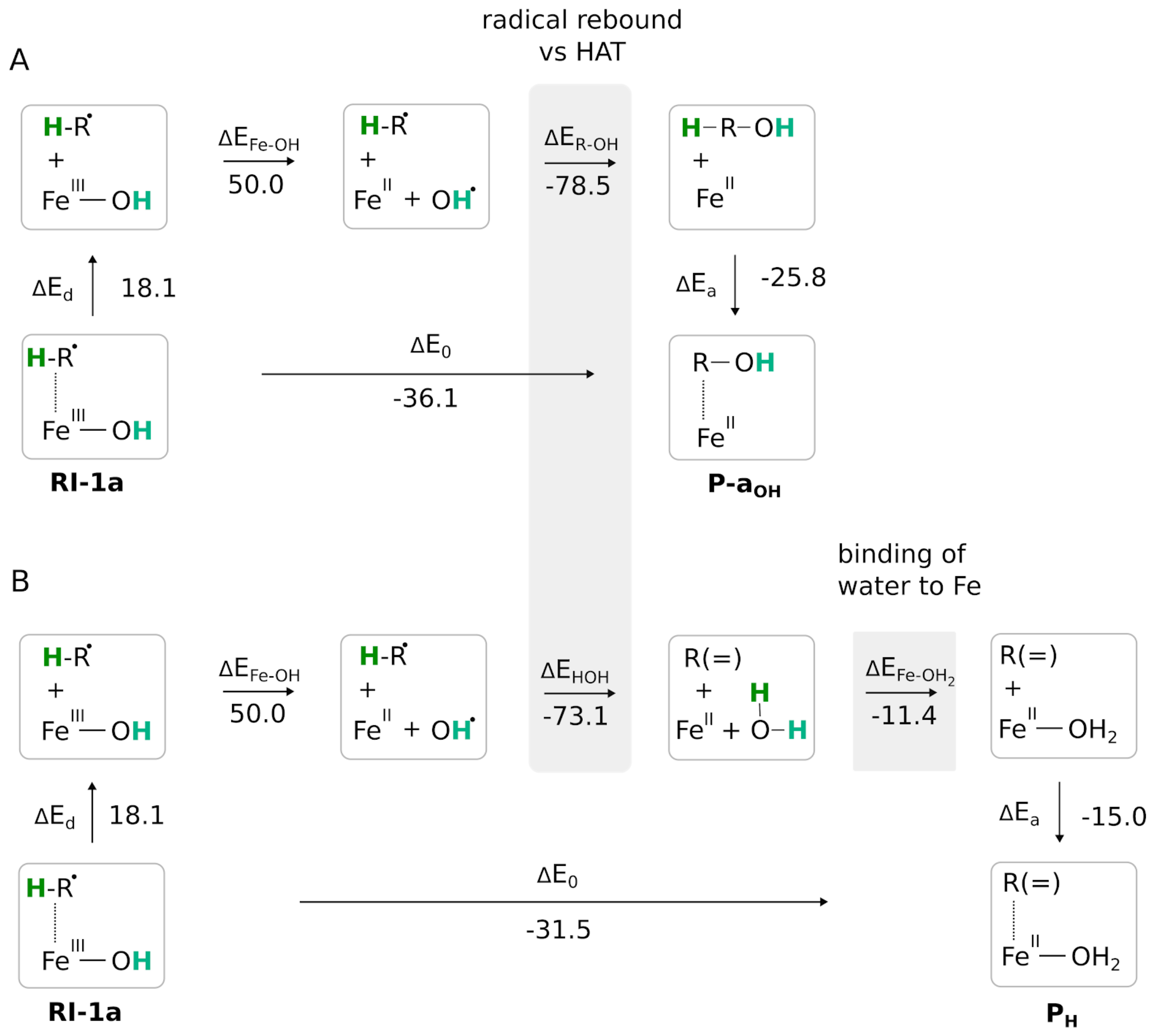

Fig. 10. The thermodynamic cycle for hydroxylation (A) and desaturation (B) in path A. The reaction energies (ΔE_0) are divided into contributions from steps analogous to ones presented in **Fig. S9.** The values are given in kcal mol^{-1} and were calculated at the B3LYP-D3/def2-TZVP// B3LYP-D3/def2-SVP level of theory.

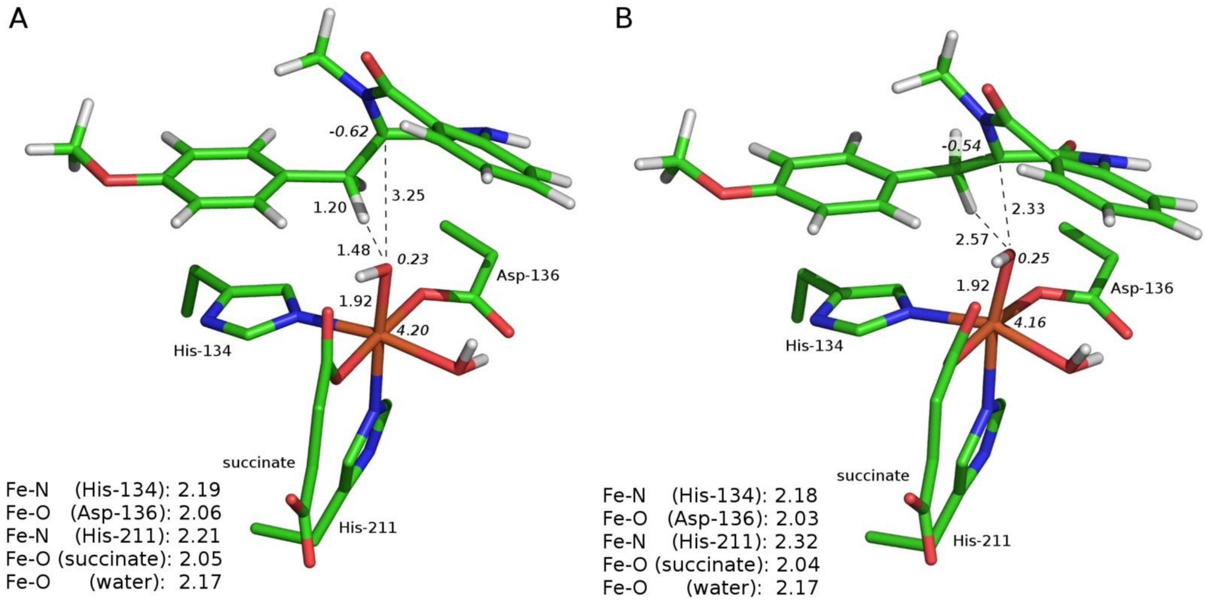

Fig. S11. The optimised structures for **TS-2a$_{\mathbf{H}}$** (A) and **TS-2a$_{\mathbf{OH}}$** (B). Distances are given in Å and spin populations larger than 0.1 are given in *italics*.

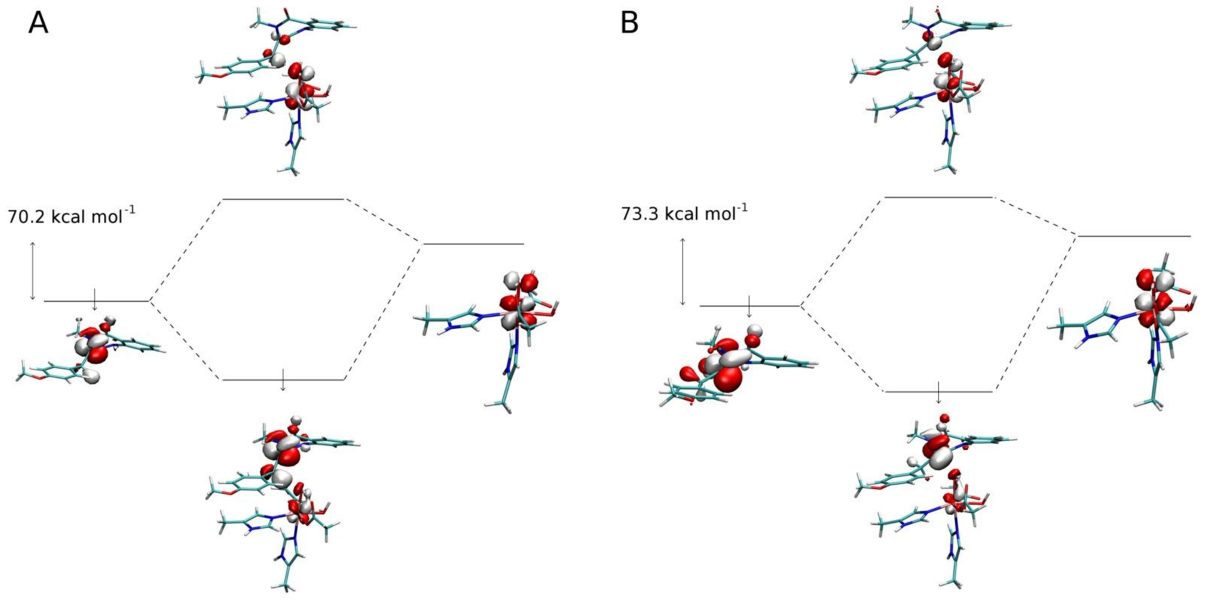

Fig. S12. Orbitals mixing in **TS-2a$_{\mathbf{H}}$** (A) and **TS-2a$_{\mathbf{OH}}$** (B). Figure rendered using VMD.

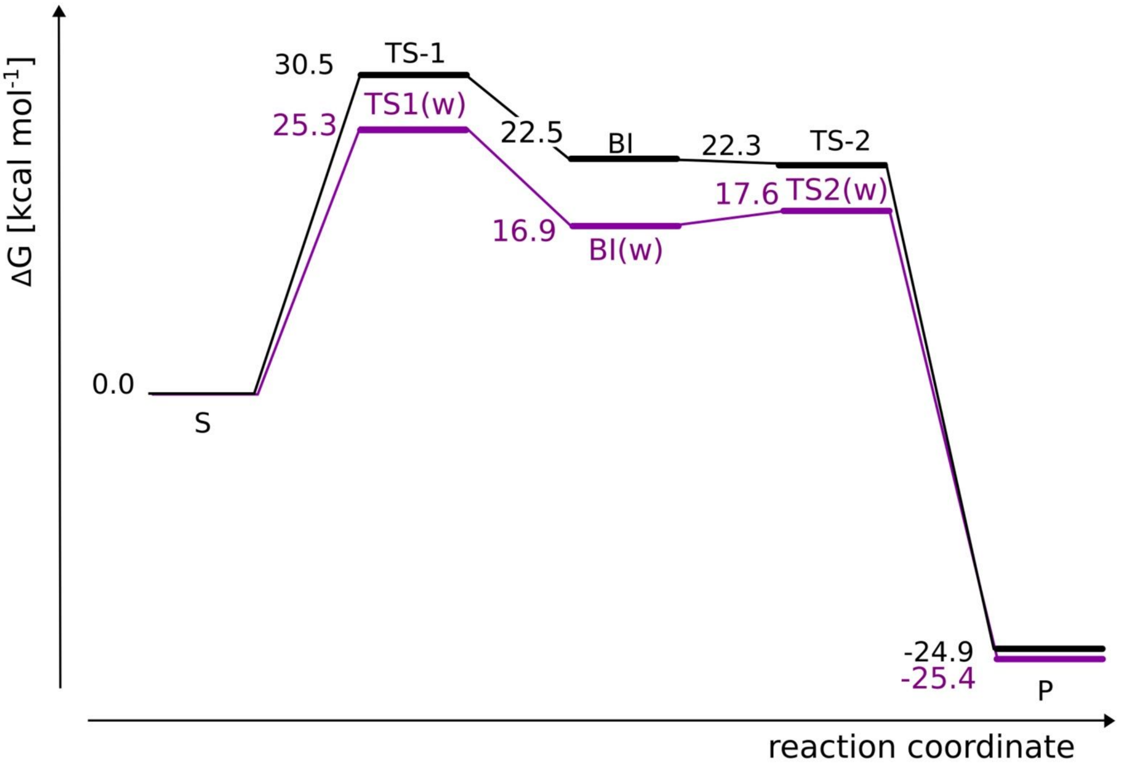

Fig. S13. Reaction profiles for rearrangement in presence of implicit water (black line), two water molecules (purple)

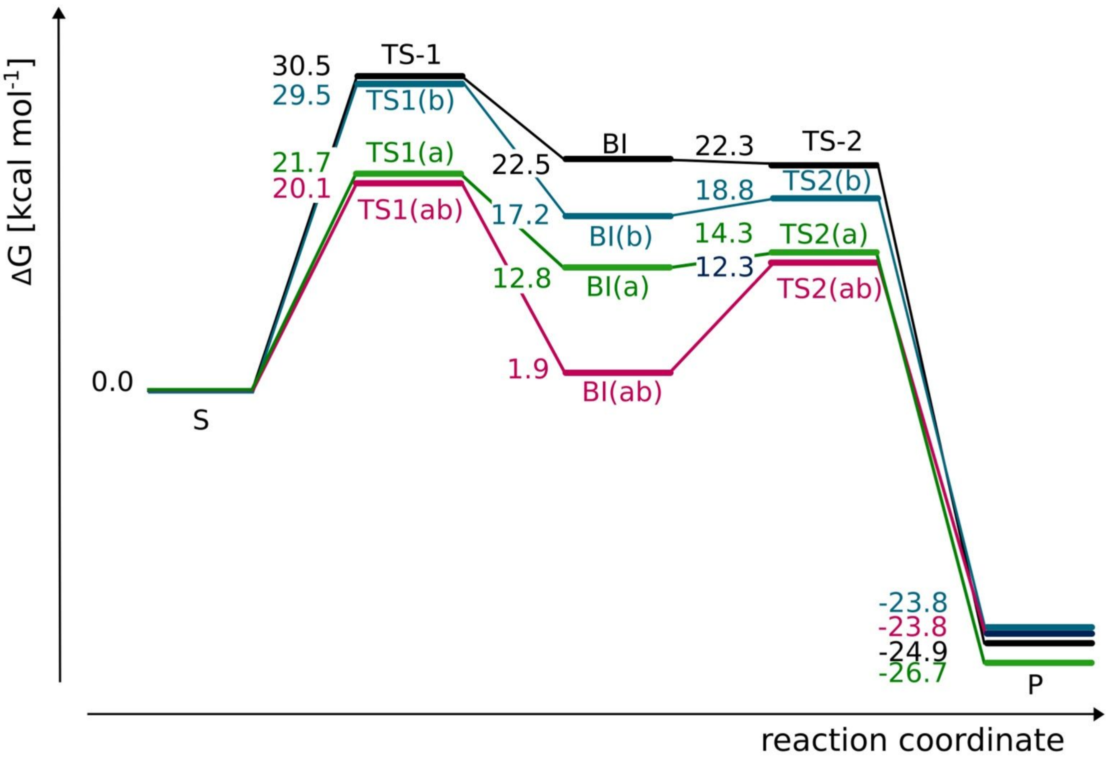

Fig. S14. Reaction profiles for rearrangement in presence of implicit water (black line), acetate (blue), acetic acid (green), acetate and acetic acid (magenta).

Table S5. Absolute energies in hartrees for stationary points in QM/MM calculations.

	B3LYP/def2-SVP mechanical embedding		B3LYP/def2-SVP mechanical embedding recalculated charges		B3LYP/def2-TZVP mechanical embedding		B3LYP/def2-TZVP electronic embedding			
	QM	ONIOM	QM	ONIOM	QM	ONIOM	QM	ONIOM	ZPE	Gibbs
**S**	-3433.74573721	-3454.1702387311	-3433.94780984	-3454.4106295699	-3436.53899698	-3457.0018152284	-3772.15655709	-3457.0140825552	18.314926	17.165129
**TS-1a**	-3433.71790949	-3454.1401425051	-3433.92484886	-3454.3845170321	-3436.51327740	-3456.9729455678	-3772.13870646	-3456.9859915545	18.308011	17.158945
**RI-1a**	-3433.76594871	-3454.1935123368	-3433.96644725	-3454.4312905151	-3436.55617796	-3457.0210202830	-3772.17416317	-3457.0328554482	18.313110	17.164931
**TS-2a$_H$**	-3433.75089426	-3454.1793768763	-3433.95088694	-3454.4223637796	-3436.53980649	-3457.0112833300	-3772.16792957	-3457.0209410701	18.310278	17.163789
**P$_H$**	-3433.81934426	-3454.2519994692	-3434.02129913	-3454.4870069644	-3436.61165581	-3457.0773584554	-3772.23417953	-3457.0857404144	18.315929	17.168172
**TS-2a$_{OH}$**	-3433.75228894	-3454.1785538689	-3433.96600926	-3454.4296857682	-3436.55198013	-3457.0156566462	-3772.17270702	-3457.0246325493	18.315114	17.169524
**P-a$_{OH}$**	-3433.81127173	-3454.2560575764	-3434.02688441	-3454.4894353703	-3436.60910341	-3457.0716512747	-3772.22015669	-3457.0798670921	18.316676	17.173225
**TS-1b** Fe(S=5/2)	-3433.71366065	-3454.1350126858	-3433.91209709	-3454.3848551589	-3436.50465411	-3456.9774121783	-3772.12083947	-3456.9873203238	18.309052	17.159719
**TS-1b** Fe(S=3/2)	-3433.70618760	-3454.1293691934	-3433.90979463	-3454.3772408269	-3436.49998918	-3456.9674353744	-3772.11247873	-3456.9791631723	18.308141	17.15928
**RI-1b**	-3433.76386130	-3454.1901113351	-3433.96494348	-3454.4337132892	-3436.55558217	-3457.0243482731	-3772.18004081	-3457.0375983541	18.312234	17.161632
**TS-2b$_H$**	-3433.75098495	-3454.1746603312	-3433.95821545	-3454.4271705795	-3436.54487201	-3457.0138271473	-3772.17346750	-3457.0258045187	18.308873	17.160996
**P$_H$**	-3433.81934426	-3454.2519994692	-3434.02129913	-3454.4870069644	-3436.61165581	-3457.0773584554	-3772.23417953	-3457.0857404144	18.315929	17.168172
**TS-2b$_{OH}$**	-3433.75315916	-3454.1755785922	-3433.95459068	-3454.4207606801	-3436.54572951	-3457.0118995021	-3772.15671015	-3457.0256089391	18.311305	17.163096
**P-b$_{OH}$**	-3433.81974654	-3454.2472231535	-3434.02345059	-3454.4908800315	-3436.60793321	-3457.0753611036	-3772.21227269	-3457.0851198092	18.315941	17.167618

Table S6. Relative energies in kcal mol^{-1} for stationary points in QM/MM calculations.

	def2-SVP mechanical embedding		def2-SVP (mechanical embedding, recalculated charges)		def2-TZVP mechanical embedding		def2-TZVP electronic embedding (EE)		corrections				
	QM	ONIOM	QM	ONIOM	QM	ONIOM	QM	ONIOM	ZPE	Gibbs	G ONIOM	G (EE) ONIOM	E+ZPE
**TS-1a**	17.5	18.9	14.4	16.4	16.1	18.1	**11.2**	**17.6**	-4.3	-3.9	14.2	**13.8**	13.3
**RI-1a**	-12.7	-14.6	-11.7	-13	-10.8	-12.1	**-11.0**	**-11.8**	-1.1	-0.1	-12.2	**-11.9**	-12.9
**TS-2a$_H$**	-3.2	-5.7	-1.9	-7.4	-0.5	-5.9	**-7.1**	**-4.3**	-2.9	-0.8	-6.8	**-5.1**	-7.2
**P$_H$**	-46.2	-51.3	-46.1	-47.9	-45.6	-47.4	**-48.7**	**-45**	0.6	1.9	-45.5	**-43.1**	-44.3
**TS-2a$_{OH}$**	-4.1	-5.2	-11.4	-12.0	-8.1	-8.7	**-10.1**	**-6.6**	0.1	2.8	-5.9	**-3.9**	-6.5
**P-a$_{OH}$**	-41.1	-53.9	-49.6	-49.5	-44.0	-43.8	**-39.9**	**-41.3**	1.1	5.1	-38.7	**-36.2**	-40.2
**TS-1b**	20.1	22.1	22.4	16.2	21.6	15.3	**22.4**	**16.8**	-3.7	-3.4	11.9	**13.4**	13.1
**TS-1b**	24.8	25.7	23.9	21.0	24.5	21.6	**27.7**	**21.9**	-4.3	-3.7	17.9	**18.2**	17.7
**RI-1b**	-11.4	-12.5	-10.8	-14.5	-10.4	-14.1	**-14.7**	**-14.8**	-1.7	-2.2	-16.3	**-17**	-16.4
**TS-2b$_H$**	-3.3	-2.8	-6.5	-10.4	-3.7	-7.5	**-10.6**	**-7.4**	-3.8	-2.6	-10.1	**-9.9**	-11.2
**P$_H$**	-46.2	-51.3	-46.1	-47.9	-45.6	-47.4	**-48.7**	**-45**	0.6	1.9	-45.5	**-43.1**	-44.3
**TS-2b$_{OH}$**	-4.7	-3.4	-4.3	-6.4	-4.2	-6.3	**-0.1**	**-7.2**	-2.3	-1.3	-7.6	**-8.5**	-9.5
**P-b$_{OH}$**	-46.4	-48.3	-47.5	-50.4	-43.3	-46.2	**-35.0**	**-44.6**	0.6	1.6	-44.6	**-43**	-43.9

Table S7. Absolute energies in hartrees for stationary points in cluster calculations. The total values are def2-TZVP energies with solvent and ZPE corrections.

	def2-SVP	def2-SVP/IEFPCM	def2-TZVP	ZPE	TOTAL
**S**	-3433.98509362	-3434.00829720	-3436.57060645	0.655527	-3435.93828303
**TS-1a**	-3433.96692199	-3433.98762685	-3436.55005600	0.648427	-3435.92233386
**RI-1a**	-3434.00043579	-3434.02200156	-3436.58511892	0.652921	-3435.95376369
**TS-2a$_H$**	-3433.98023347	-3434.00163526	-3436.56360356	0.647587	-3435.93741835
**P$_H$**	-3434.05086560	-3434.07329396	-3436.63525803	0.652942	-3436.00474439
**TS-2a$_{OH}$**	-3433.99832565	-3434.02100350	-3436.58571538	0.652495	-3435.95589823
**P-a$_{OH}$**	-3434.06838931	-3434.08957132	-3436.64277703	0.654857	-3436.00910204
**TS-1b**	-3433.95325239	-3433.97738894	-3436.54173102	0.647812	-3435.91805557
**RI-1b**	-3434.00069829	-3434.02371321	-3436.58622672	0.651968	-3435.95727364
**TS-2b$_H$**	-3433.99016495	-3434.01319313	-3436.57201160	0.647414	-3435.94762578
**P$_H$**	-3434.05086560	-3434.07329396	-3436.63525803	0.652942	-3436.00474439
**TS-2b$_{OH}$**	-3433.98257504	-3434.00674096	-3436.56774546	0.651966	-3435.93994538
**P-b$_{OH}$**	-3434.05066706	-3434.07353376	-3436.63085732	0.656298	-3435.99742548

Table S8. Relative energies in kcal mol^{-1} for stationary points in cluster calculations. The total values are def2-TZVP energies with solvent and ZPE corrections.

	def2-SVP	SCRF	def2-TZVP	ZPE	TOTAL
**TS-1a**	11.40	12.97	12.90	-4.46	**10.01**
**RI-1a**	-9.63	-8.60	-9.11	-1.64	**-9.71**
**TS-2a$_H$**	3.05	4.18	4.39	-4.98	**0.54**
**P$_H$**	-41.27	-40.79	-40.57	-1.62	**-41.71**
**TS-2a$_{OH}$**	-8.30	-7.97	-9.48	-1.90	**-11.05**
**P-a$_{OH}$**	-52.27	-51.00	-45.29	-0.42	**-44.44**
**TS-1b**	19.98	19.40	18.12	-4.84	**12.69**
**RI-1b**	-9.79	-9.67	-9.80	-2.23	**-11.92**
**TS-2b$_H$**	-3.18	-3.07	-0.88	-5.09	**-5.86**
**P$_H$**	-41.27	-40.79	-40.57	-1.62	**-41.71**
**TS-2b$_{OH}$**	1.58	0.98	1.80	-2.23	**-1.04**
**P-b$_{OH}$**	-41.15	-40.94	-37.81	0.48	**-37.11**

Table S9. Absolute energies in hartrees for stationary points in the nonenzymatic rearrangement path. The total values are 6-311G(d,p)/IEFPCM energies with ZPE or Gibbs free energy corrections. **P(1)** stands for 4'-methoxyviridicatin, **P(2)** for methylisocyanate.

**Solvent modelled as PCM**						
	6-31G/IEFPCM	6-311G(d,p)/IEFPCM	ZPE	Gibbs	TOTAL/ZPE	TOTAL/Gibbs
**S**	-1105.71744799	-1106.31033682	0.315663	0.26537	-1105.99467382	-1106.04496682
**TS1**	-1105.67443922	-1106.26134751	0.313327	0.265038	-1105.94802051	-1105.99630951
**BI**	-1105.68428423	-1106.27425402	0.314271	0.26512	-1105.95998302	-1106.00913402
**TS2**	-1105.6808054	-1106.27305762	0.312785	0.263711	-1105.96027262	-1106.00934662
**P(1)**	-897.797217705	-898.264197695	0.260327	0.214406	-898.003870695	-898.049791695
**P(2)**	-207.925789119	-208.057625771	0.050338	0.022715	-208.007287771	-208.034910771
**P**	-1105.72300682	-1106.321823466	0.310665	0.237121	-1106.01115846	-1106.08470246
**Acetic acid + acetate**						
	6-31G/IEFPCM	6-311G(d,p)/IEFPCM	ZPE	Gibbs	TOTAL/ZPE	TOTAL/Gibbs
**S**	-1563.33370184	-1564.20735428	0.42827	0.357708	-1563.77908428	-1563.84964628
**TS1**	-1563.31017066	-1564.17436265	0.424167	0.356769	-1563.75019565	-1563.81759365
**BI**	-1563.32617042	-1564.19317298	0.424367	0.354754	-1563.76880598	-1563.83841898
**TS2**	-1563.31345912	-1564.18527484	0.424268	0.355154	-1563.76100684	-1563.83012084
**P(1)**	-1355.41339304	-1356.16186143	0.37293	0.309265	-1355.78893143	-1355.85259643
**P(2)**	-207.925789119	-208.057625771	0.050338	0.022715	-208.007287771	-208.034910771
**P**	-1563.33918215	-1564.21948720	0.423268	0.33198	-1563.79621920	-1563.88750720
**Acetate**						
	6-31G/IEFPCM	6-311G(d,p)/IEFPCM	ZPE	Gibbs	TOTAL/ZPE	TOTAL/Gibbs
**S**	-1334.29209761	-1335.02332242	0.365154	0.303649	-1334.65816842	-1334.71967342
**TS1**	-1334.25169678	-1334.97638396	0.362521	0.303702	-1334.61386296	-1334.67268196
**BI**	-1334.26233633	-1334.99603441	0.361722	0.303842	-1334.63431241	-1334.69219241
**TS2**	-1334.25988843	-1334.99069861	0.361532	0.301041	-1334.62916661	-1334.68965761
**P(1)**	-1126.37080815	-1126.97621907	0.309924	0.253594	-1126.66629507	-1126.72262507
**P(2)**	-207.925789119	-208.057625771	0.050338	0.022715	-208.007287771	-208.034910771
**P**	-1334.296597269	-1335.03384484	0.360262	0.276309	-1334.67358284	-1334.75753584
**Acetic acid**						
	6-31G/IEFPCM	6-311G(d,p)/IEFPCM	ZPE	Gibbs	TOTAL/ZPE	TOTAL/Gibbs
**S**	-1334.75823717	-1335.4936819	0.378907	0.319126	-1335.1147749	-1335.1745559
**TS1**	-1334.73090112	-1335.45782917	0.375423	0.317901	-1335.08240617	-1335.13992817
**BI**	-1334.74231953	-1335.47195767	0.376329	0.317832	-1335.09562867	-1335.15412567
**TS2**	-1334.73476102	-1335.46820272	0.375221	0.316418	-1335.09298172	-1335.15178472
**P(1)**	-1126.83915658	-1127.44972537	0.323582	0.267557	-1127.12614337	-1127.18216837
**P(2)**	-207.925789119	-208.057625771	0.050338	0.022715	-208.007287771	-208.034910771
**P**	-1334.764945699	-1335.50735114	0.37392	0.290272	-1335.13343114	-1335.21707914
**Water molecules**						
	6-31G/IEFPCM	6-311G(d,p)/IEFPCM	ZPE	Gibbs	TOTAL/ZPE	TOTAL/Gibbs
**S**	-1258.54855427	-1259.25145034	0.363816	0.306135	-1258.88763434	-1258.94531534
**TS1**	-1258.51348951	-1259.20891905	0.360975	0.303876	-1258.84794405	-1258.90504305
**BI**	-1258.52584456	-1259.2239173	0.362445	0.305523	-1258.8614723	-1258.9183943

**TS2**	-1258.51815754	-1259.22012148	0.360707	0.302909	-1258.85941448	-1258.91721248
**P(1)**	-1050.62588085	-1051.20286414	0.307653	0.252027	-1050.89521114	-1050.95083714
**P(2)**	-207.925789119	-208.057625771	0.050338	0.022715	-208.007287771	-208.034910771
**P**	-1258.551669969	-1259.26048991	0.357991	0.274742	-1258.90249891	-1258.98574791

Ascorbic acid+ascorbate

	6-31G/IEFPCM	6-311G(d,p)/IEFPCM	ZPE	Gibbs	TOTAL/ZPE	TOTAL/Gibbs
**S**	-2474.49554199	-2475.91972022	0.604853	0.523725	-2475.31486722	-2475.39599522
**TS1**	-2474.48950028	-2475.90714704	0.602437	0.525397	-2475.30471004	-2475.38175004
**BI**	-2474.49950458	-2475.92063282	0.603172	0.525213	-2475.31746082	-2475.39541982
**TS2**	-2474.48216502	-2475.90604519	0.600399	0.520628	-2475.30564619	-2475.38541719
**P(1)**	-2266.58157243	-2267.87999316	0.549521	0.47519	-2267.33047216	-2267.40480316
**P(2)**	-207.925789119	-208.057625771	0.050338	0.022715	-208.007287771	-208.034910771
**P**	-2474.507361549	-2475.93761893	0.599859	0.497905	-2475.33775993	-2475.43971393

Ascorbic acid

	6-31G/IEFPCM	6-311G(d,p)/IEFPCM	ZPE	Gibbs	TOTAL/ZPE	TOTAL/Gibbs
**S**	-1790.31933946	-1791.3353119	0.465813	0.398393	-1790.8694989	-1790.9369189
**TS1**	-1790.3090947	-1791.31636405	0.463933	0.399813	-1790.85243105	-1790.91655105
**BI**	-1790.32270653	-1791.33291877	0.465578	0.401354	-1790.86734077	-1790.93156477
**TS2**	-1790.31091902	-1791.32075634	0.462991	0.399315	-1790.85776534	-1790.92144134
**P(1)**	-1582.40781382	-1583.29560989	0.410787	0.349077	-1582.88482289	-1582.94653289
**P(2)**	-207.925789119	-208.057625771	0.050338	0.022715	-208.007287771	-208.034910771
**P**	-1790.333602939	-1791.35323566	0.461125	0.371792	-1790.89211066	-1790.98144366

Ascorbate

	6-31G/IEFPCM	6-311G(d,p)/IEFPCM	ZPE	Gibbs	TOTAL/ZPE	TOTAL/Gibbs
**S**	-1789.87977861	-1790.88738317	0.453679	0.387808	-1790.43370417	-1790.49957517
**TS1**	-1789.83909958	-1790.84112264	0.450898	0.386062	-1790.39022464	-1790.45506064
**BI**	-1789.8476006	-1790.85296978	0.451556	0.385769	-1790.40141378	-1790.46720078
**TS2**	-1789.84191334	-1790.85005088	0.449676	0.382709	-1790.40037488	-1790.46734188
**P(1)**	-1581.95540944	-1582.83857126	0.397704	0.335079	-1582.44086726	-1582.50349226
**P(2)**	-207.925789119	-208.057625771	0.050338	0.022715	-208.007287771	-208.034910771
**P**	-1789.881198559	-1790.89619703	0.448042	0.357794	-1790.44815503	-1790.53840303

Table S10. Relative energies in kcal mol^{-1} for stationary points in the nonenzymatic rearrangement path. The total values are 6-311G(d,p)/IEFPCM energies with ZPE or Gibbs free energy corrections.

**Solvent modelled as PCM**						
	6-31G/IEFPCM	6-311G(d,p)/IEFPCM	ZPE	Gibbs	**TOTAL/ZPE**	**TOTAL/Gibbs**
**TS1**	26.99	30.74	-1.47	-0.21	**29.28**	**30.53**
**BI**	20.81	22.64	-0.87	-0.16	**21.77**	**22.49**
**TS2**	22.99	23.39	-1.81	-1.04	**21.59**	**22.35**
**P**	-3.49	-7.21	-3.14	-17.73	**-10.34**	**-24.93**
**Acetic acid + acetate**						
	6-31G/IEFPCM	6-311G(d,p)/IEFPCM	ZPE	Gibbs	**TOTAL/ZPE**	**TOTAL/Gibbs**
**TS1**	14.77	20.70	-2.57	-0.59	**18.13**	**20.11**
**BI**	4.31	3.67	-1.95	-1.72	**1.72**	**1.95**
**TS2**	12.70	13.86	-2.51	-1.60	**11.34**	**12.25**
**P**	-3.44	-7.61	-3.14	-16.14	**-10.75**	**-23.76**
**Acetate**						
	6-31G/IEFPCM	6-311G(d,p)/IEFPCM	ZPE	Gibbs	**TOTAL/ZPE**	**TOTAL/Gibbs**
**TS1**	25.35	29.45	-1.65	0.03	**27.80**	**29.49**
**BI**	18.68	17.12	-2.15	0.12	**14.97**	**17.24**
**TS2**	20.21	20.47	-2.27	-1.64	**18.20**	**18.84**
**P**	-2.82	-6.60	-3.07	-17.16	**-9.67**	**-23.76**
**Acetic acid**						
	6-31G/IEFPCM	6-311G(d,p)/IEFPCM	ZPE	Gibbs	**TOTAL/ZPE**	**TOTAL/Gibbs**
**TS1**	17.15	22.50	-2.19	-0.77	**20.31**	**21.73**
**BI**	9.99	13.63	-1.62	-0.81	**12.01**	**12.82**
**TS2**	14.73	15.99	-2.31	-1.70	**13.68**	**14.29**
**P**	-4.21	-8.58	-3.13	-18.11	**-11.71**	**-26.68**
**Water molecules**						
	6-31G/IEFPCM	6-311G(d,p)/IEFPCM	ZPE	Gibbs	**TOTAL/ZPE**	**TOTAL/Gibbs**
**TS1**	22.00	26.69	-1.78	-1.42	**24.91**	**25.27**
**BI**	14.25	17.28	-0.86	-0.38	**16.42**	**16.89**
**TS2**	19.07	19.66	-1.95	-2.02	**17.71**	**17.63**
**P**	-1.96	-5.67	-3.66	-19.70	**-9.33**	**-25.37**
**Ascorbic acid+ascorbate**						
	6-31G/IEFPCM	6-311G(d,p)/IEFPCM	ZPE	Gibbs	**TOTAL/ZPE**	**TOTAL/Gibbs**
**TS1**	3.79	7.89	-1.52	1.05	**6.37**	**8.94**
**BI**	-2.49	-0.57	-1.05	0.93	**-1.63**	**0.36**
**TS2**	8.39	8.58	-2.79	-1.94	**5.79**	**6.64**
**P**	-7.42	-11.23	-3.13	-16.20	**-14.37**	**-27.43**
**Ascorbic acid**						
	6-31G/IEFPCM	6-311G(d,p)/IEFPCM	ZPE	Gibbs	**TOTAL/ZPE**	**TOTAL/Gibbs**
**TS1**	6.43	11.89	-1.18	0.89	**10.71**	**12.78**
**BI**	-2.11	1.50	-0.15	1.86	**1.35**	**3.36**
**TS2**	5.28	9.13	-1.77	0.58	**7.36**	**9.71**

**P**	-8.95	-16.69	-2.94	-16.69	**-14.19**	**-27.94**
**Ascorbate**						
	6-31G/IEFPCM	6-311G(d,p)/IEFPCM	ZPE	Gibbs	**TOTAL/ZPE**	**TOTAL/Gibbs**
**TS1**	25.53	29.03	-1.75	-1.10	**27.28**	**27.93**
**BI**	20.19	21.59	-1.33	-1.28	**20.26**	**20.32**
**TS2**	23.76	23.43	-2.51	-3.20	**20.91**	**20.23**
**P**	-0.89	-18.83	-3.54	-18.83	**-9.07**	**-24.36**
